# Newcomer to the Calixarene Family: Synthesis and Characterization of Selenacalix[4]arene

**DOI:** 10.1021/acs.orglett.5c04133

**Published:** 2025-12-09

**Authors:** Michal Churý, Tadeáš Petrů, Radek Staník, Jan Sýkora, Václav Eigner, Pavel Lhoták

**Affiliations:** † Department of Organic Chemistry, 52735 University of Chemistry and Technology Prague (UCTP), Technická 5, 166 28, Prague 6, Czech Republic; ‡ Department of Analytical Chemistry, 52735 University of Chemistry and Technology Prague (UCTP), Technická 5, 166 28, Prague 6, Czech Republic; § Institute of Physics AS CR, v.v.i., Na Slovance 1999/2, 182 21, Prague 8, Czech Republic

## Abstract

In this work, we describe a general and efficient fragment condensation method for the synthesis of monoselenacalix[4]­arene as a new member of the broad calixarene family. The key fragments were obtained from the starting *p*-*tert*-butylphenol by reaction with *in situ* generated SeCl_2_ or from its formyl derivative by reaction with SeO_2_ in pyridine. The availability of both fragments allowed us to test two different independent macrocyclization routes, leading to the final selenacalixarene in good yields. The first successful attempts to immobilize the new system were made, and the basic dynamics of the macrocycles in solution were studied using VT NMR techniques. The selenium analogue of calix[4]­arene was successfully immobilized in the *cone* conformation, suggesting its potential use in supramolecular chemistry.

## Graphical abstract

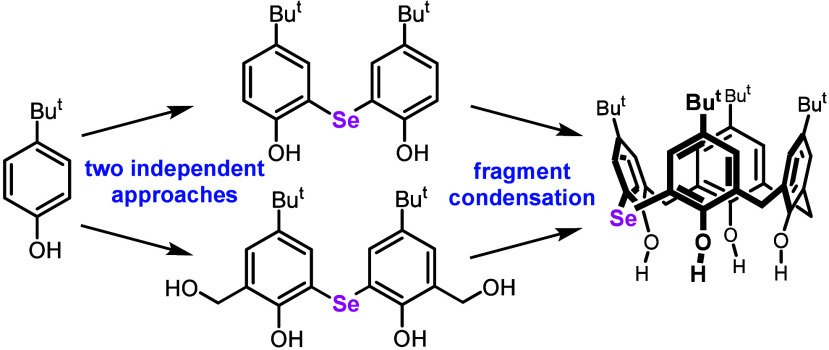

Calix­[*n*]­arenes **I** (Figure 1) represent a well-known family^1^ of macrocycles, which are among the cornerstones of contemporary supramolecular chemistry.^2^ Due to their large-scale preparation and well-established chemistry, these pre-organized systems found a wide range of possible applications.^3^

**Figure 1:**
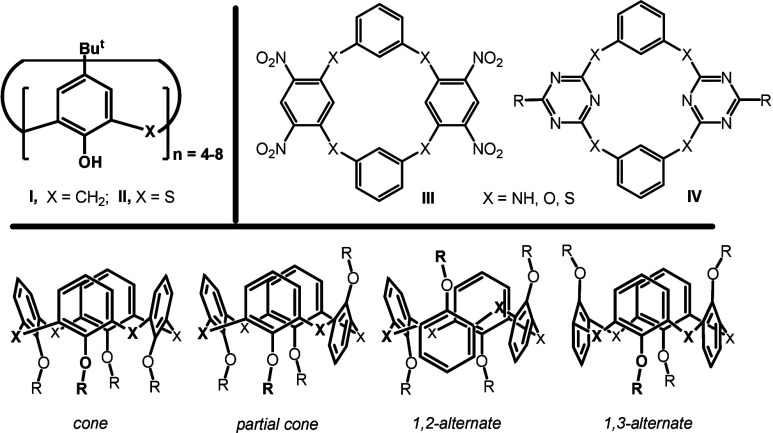
Calix­[*n*]­arenes **I** versus thiacalix­[*n*]­arenes **II**, examples of some selected “heteracalixarenes” **III** and **IV**, and 3D structures of basic (thia)­calix[4]­arene conformers (atropisomers).

The well-established chemistry of classical calixarenes has been significantly revitalized by the introduction of sulfur instead of the usual CH_2_ bridging units.^4^ The so-called thiacalixarenes **II** have very different properties compared to the original classical calixarenes. The presence of the four sulfur atoms in thiacalix[4]­arene induces dramatic changes not only in the complexation behavior and conformational preferences but also in the basic chemistry of such compounds.^5^ Furthermore, the introduction of just one or two heteroatoms confers surprising properties on these mixed-bridged systems.^6^

Many examples of aromatic macrocycles with heteroatom bridges can be found in the literature. This numerous and structurally very heterogeneous group of compounds can be demonstrated, for example, by formulas **III** and **IV** in Figure 1.^7^ The preparation of these systems is usually based on aromatic nucleophilic substitution, and they can carry different heteroatoms (such as NH, O, S, etc.) or different aromatic nuclei. From the perspective of supramolecular chemistry, these compounds, often called calixarenes, offer a number of very interesting properties. However, the absence of hydroxyl groups does not allow for simple immobilization (usually by alkylation of OH groups) of these macrocycles in the chosen conformation, which is probably the greatest advantage and the most attractive feature of true calix[4]­arene or thiacalix[4]­arene systems (such as **I** and **II**; *n* = 4).

The well-defined three-dimensional structures (*cone*, *partial cone*, *1,2-alternate*, or *1,3-alternate* in Figure 1) of (thia)­calix[4]­arenes and their close relatives, such as homooxacalix[4]­arenes,^8^ can then be advantageously used as molecular scaffolds and/or useful building blocks in the construction of more sophisticated supramolecular systems. Since the presence of a heteroatom is a key prerequisite for completely new or substantially altered properties of the parent macrocycles, it would be interesting to prepare true calixarene systems containing other heteroatoms. This work describes the synthesis and basic characterization of such mixed-bridged calix[4]­arenes containing a selenium atom.

Organoselenium compounds attract great attention not only in medicinal chemistry^9^ but have also found applications in catalysis, materials chemistry, and organic synthesis.^10^ The literature offers a wealth of different approaches^11^ to the preparation of diaryl selenides (Scheme 1), which differ in the source of selenium or the starting arenes. Thus, the reaction of haloarenes (usually iodo- or bromo-) and elemental selenium (a^12^ or b^13^), tributylstannyl selenides (g and h),^14^ or arylbenzyl selenide (i)^15^ can be used to construct diaryl selenides. Similarly, areneboronic acids (n^16^ and m^17^), aromatic amines (l),^18^ arenediazonium salts (k),^19^ and arylhydrazines^20^ can be combined with various selenium-based reaction partners, often under transition metal catalysis. Another option is a direct substitution of the arenes (C–H selenation) with a suitable selenium reagent, such as aryl selenium chloride (c),^21^ diaryl selenide (d),^22^ SeCl_2_ (e),^23^ or SeO_2_ (f).^24^

**Scheme 1:**
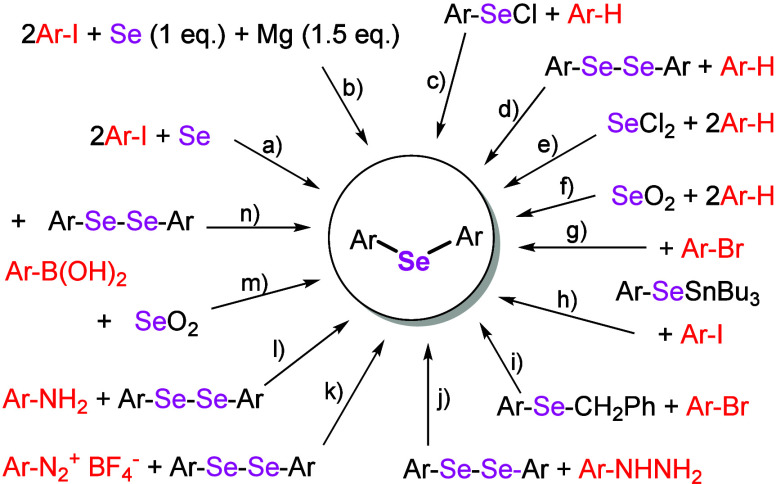
Selected Syntheses of Diaryl Selenides

Since calixarenes are obtained^1^ from simple starting compounds, we tried to follow this approach also in the synthesis of selenium analogues, so that the synthesis would be sufficiently robust, simple, and scalable. In this context, although macrocycles can be found in the literature that are referred to as calixarenes and carry selenium as a bridge (selenacalix[3]­triazines)^25^ or part of a bridge (homodiselenacalix[4]­arene),^26^ these are again not true calixarenes. It is clear that the current state of the art does not allow for the direct synthesis of calix[4]­arenes bearing all Se bridges. Therefore, we decided to apply the so-called fragment condensation of appropriately arranged building blocks (bisphenols linked by a selenium bridge), which are, however, still unknown.

Since all our efforts to utilize the reaction of iodine-substituted compounds [4-(*tert*-butyl)-2-iodophenol or 4-(*tert*-butyl)-2-iodo-1-methoxybenzene] with Se (path a, Scheme 1) completely failed, an attempt was made to synthesize bisphenol **2** by reacting the starting *p*-*tert*-butylphenol **1** with SeO_2_ in hydrochloric acid (Scheme 2). The rather diverse reaction mixture (TLC) showed signals in the ^1^H NMR spectrum (CDCl_3_), indicating the expected splitting pattern of the product. However, HRMS confirmed the presence of other byproducts with identical splitting patterns in ^1^H NMR (diselenide and biphenyl analogues), accompanied by several different tris-phenols, all with almost identical *R*_f_ values. Similarly, the reaction of SeO_2_ in pyridine^27^ led to a complex reaction mixture containing the desired product, which, however, could not be purified by conventional chromatographic separations.

**Scheme 2:**
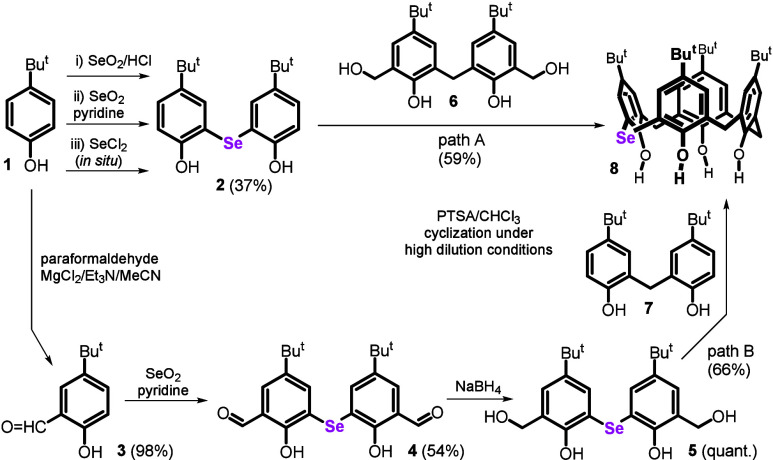
Synthesis of Monoselenacalix[4]­arene

SeCl_2_ as a potential reagent for electrophilic substitution is not commercially available due to its instability at laboratory temperature, but it can be generated *in situ* as a complex with THF,^28^ which is stable for hours. Thus, elemental selenium and SO_2_Cl_2_ reacted at room temperature and finally diluted by THF. This solution was then added to **1**, and the reaction mixture was stirred overnight. This procedure led to product **2** in 41% yield after chromatography. The HRMS ESI^–^ analysis of **2** showed a signal at *m*/*z* 377.1024, which was in accordance with the [M – H]^−^ anion predicted for the product (*m*/*z* 377.1025). The ^77^Se NMR spectrum (95 MHz, CDCl_3_) supported the presence of diaryl selenide (δ 230.1 ppm).

Experiments with SeO_2_ suggested that there may be a problem with possible multiple reactivity at the 2 and 6 positions of the phenolic unit. We attempted to circumvent this problem by introducing a formyl group, which would serve as a protecting group for this position and at the same time allow the hydroxymethyl derivative to be obtained. Reaction of **1** with MgCl_2_ (Scheme 2), triethylamine, and paraformaldehyde gave product **3** in excellent yield (98%).^29^ While the reaction with SeCl_2_ did not give any product as the system is too deactivated by the formyl group (and so did the reaction with SeO_2_ in aqueous HCl), the reaction with SeO_2_ in pyridine yielded the expected product **4** in an acceptable yield (42%). Subsequent reduction with NaBH_4_ gave hydroxymethylated bisphenol **5** in 98% yield. The two independent routes to the synthesis of selenium bisphenols **2** and **5** represent a significant innovation compared to the chemistry of sulfur analogues,^30^ where the sulfur derivative of type **5** is only available by hydroxymethylation of the corresponding sulfur analogue of compound **2**.

An initial attempt at macrocyclization of blocks **2** and **6** (path A) under high dilution conditions (toluene, 100 °C/16 h, and PTSA) resulted in the isolation of product **8** in only 8% yield. Surprisingly, a reaction similar to that with blocks **5** and **7** (path B) did not provide the macrocycle at all. A thorough analysis of the reaction mixture using HRMS combined with preparative TLC led to the detection of a wide range of products, indicating the reaction of toluene with the used building blocks and at the same time their unwanted cleavage (see Figures S1–S4 and Tables S1–S4). Lowering the reaction temperature and time (65 °C/5 h) and replacing toluene with chloroform then led to the smooth formation of monoselena derivative **8** (59% yield) for route A (**2** + **6**). Similarly, the reaction of blocks **5** and **7** (route B) gave a target macrocycle **8** in 66% yield.

The ^1^H NMR spectrum (400 MHz, CDCl_3_, 298 K) reflects the presence of a symmetry plane passing through selenium and the opposite CH_2_ bridge. As a result, four doublets in the aromatic region (7.54, 7.22, 7.08, and 7.05 ppm) with typical *meta* splitting (*J* ≈ 2.5 Hz) and two singlets of *tert*-butyl groups at 1.20 and 1.21 ppm can be found. At the same time, two very broad unresolved signals of CH_2_ bridges at 3.55 ppm (equatorial C–H bonds) and 4.25 ppm (axial C–H bonds) are visible in the spectrum. All these features suggest (together with the OH group signal at 9.97 ppm) that compound **8** adopts a *cone* conformation in solution.

The final structural proof of **8** was performed using single-crystal X-ray diffraction analysis. The compound crystallized in a tetragonal crystal system, with space group *P*4/*n*, and adopted the *cone* conformation held together by a circular hydrogen bond array on the lower rim (see Figures S45 and S46). Unfortunately, the symmetry of the macrocycle was too high to keep only one orientation of the molecule within the crystal lattice. As a result, the selenium bridge was statistically distributed across all four positions, with an occupancy of 25%. The same is true for the remaining three methylene groups with an occupancy of 75% bridges.

The selenacalixarene *cone* conformers are connected to each other by η^6^-chalcogen bonding between the Se bridge and the phenolic moiety of the neighbor molecule. Regrettably, the disorder in the structure does not allow us to determine whether this interaction leads to the formation of dimers, tetramers, or other more complex arrangements within the structure (see Figures S47 and S48). In the absence of a Se bridging atom, the chalcogen bond is replaced by much weaker London interactions of CH_2_.

The basic prerequisite for the successful use of a new macrocycle in supramolecular applications is knowledge of its conformational behavior. The broad signals of methylene bridges in the ^1^H NMR spectrum of compound **8** (see above) indicate the dynamic behavior of the macrocycle in solution. This phenomenon is typical for calix[4]­arenes with an unsubstituted lower rim (free OHs) and indicates an interconversion of the two *cone* conformations, which is manifested as a chemical exchange of the diastereotopic CH_2_ hydrogen atoms.

This dynamic behavior was further studied by variable-temperature (VT) ^1^H NMR (500 MHz) performed in C_2_D_2_Cl_4_ (Figure 2). Increasing the temperature led to a collapse of axial and equatorial CH_2_ resonances into a single signal at 3.95 ppm with the coalescence temperature at around 330 K, while decreasing the temperature provided sharp signals with the geminal interaction constant of 14 Hz. The ^1^H NMR spectra obtained in the range of 263–353 K were then analyzed by dynamic NMR models (dNMR) in the TopSpin software package, allowing determination of the rate constant *k* for each temperature. Subsequently, the activation free energy was determined using the Eyring equations. The calculated value of the energy barrier (Δ*G*_300 K_^#^ = 64.7 kJ mol^–1^) is surprisingly similar to that of *tert*-butylcalix­[4]­arene **I** in CDCl_3_ (65.7 kJ mol^–1^).^31^

**Figure 2:**
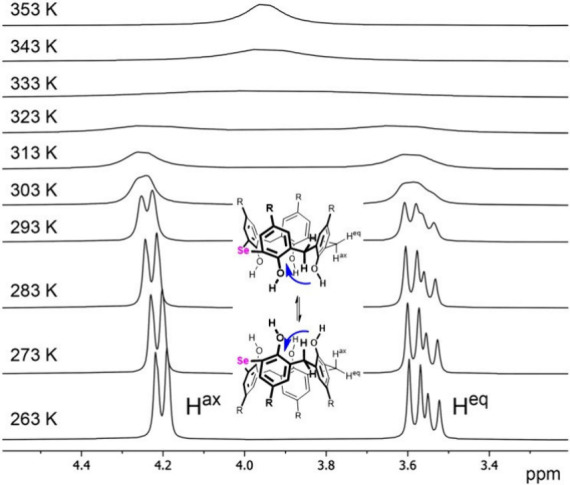
Partial VT ^1^H NMR spectra (C_2_D_2_Cl_4_, 500 MHz) of **8a** showing the CH_2_ bridge area.

A dynamic behavior of phenolic OH groups corresponding to the flip-flop motion (the change of direction) of the circular hydrogen bond array was investigated by ^1^H NMR in CD_2_Cl_2_. The two original signals in the ratio 1:1 get broadened with a decreasing temperature and finally split below 215 K into three resonances in the mutual ratio 2:1:1 (Figure 3). The line shape fitting in the temperature range of 193–253 K using dNMR provided the activation free energy Δ*G*_300 K_^#^ of 45.1 kJ mol^–1^. A comparison to the barrier (Δ*G*_300 K_^#^ = 47.6 kJ mol^–1^)^32^ reported for classical *tert*-butylcalix­[4]­arene **I** suggests that the introduction of the selenium atom leads to a weakening of the circular hydrogen bond. Qualitatively, the same conclusion can be reached by comparing the chemical shifts of the phenolic OH groups in CDCl_3_ at 298 K (10.3 ppm for **I** vs 9.97 ppm for **8**).

**Figure 3:**
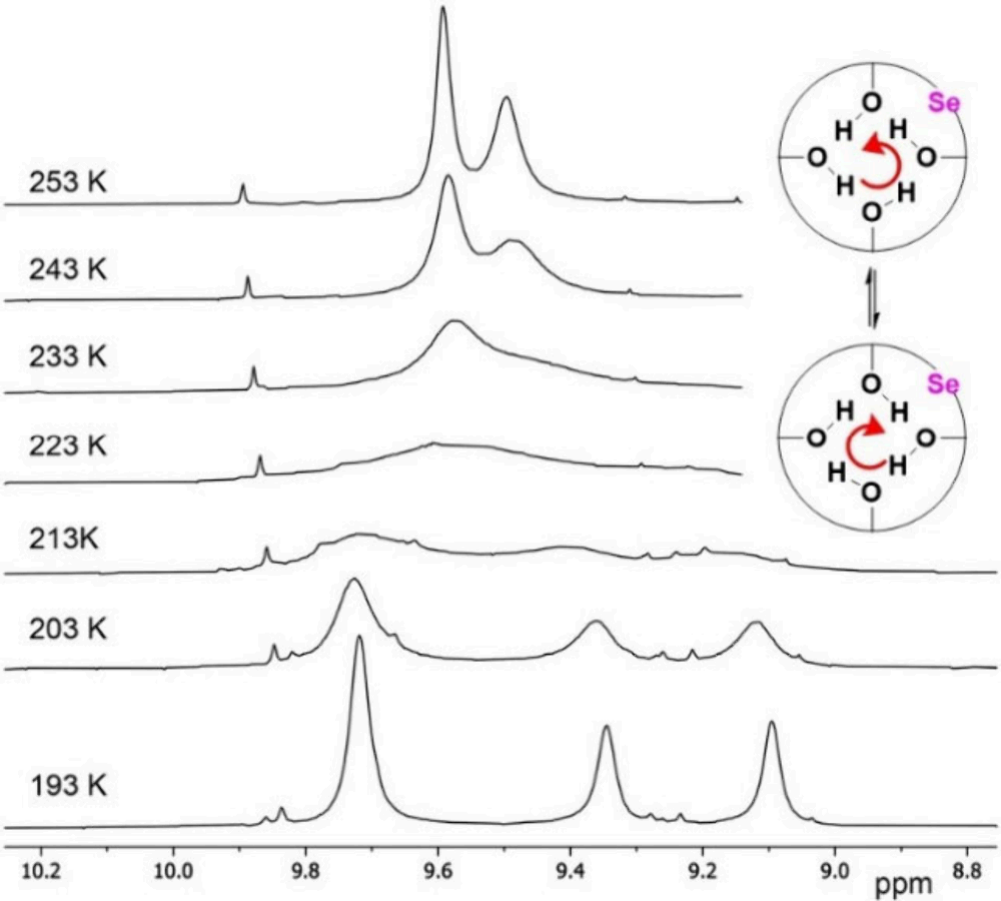
Partial ^1^H NMR spectrum (CD_2_Cl_2_, 500 MHz) of **8** in the range of 193–253 K (flip-flop motion of the OH groups).

As already mentioned, perhaps the greatest advantage of calix[4]­arenes is the possibility of fixing the molecule by simple alkylation of the lower rim. A preliminary study with selenium analogue **8** showed that immobilization could be achieved by alkylation with PrI in the presence of NaH in DMF (Scheme 3). Under these circumstances, no alkylation of the selenium atom (potentially very strong nucleophile) was observed. The corresponding *cone* conformer **9** was obtained after preparative TLC (silica gel) as the only isolable alkylation product (49% yield).

**Scheme 3:**
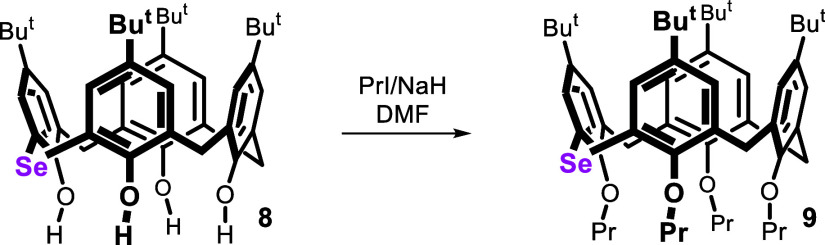
Alkylation of Monoselenacalix[4]­arene **8**

The ^1^H NMR spectrum of **9** (CDCl_3_, 500 MHz) showed four doublets in the aliphatic part of the spectrum (3.15 and 3.18 ppm for equatorial CH bonds and 4.46 and 4.51 ppm for axial CH bonds), with typical geminal coupling constants (*J* ≈ 12.6 Hz) corresponding to the signals of the bridging CH_2_ moieties in the *cone* conformation (see Figure S29). The presence of two singlets of *tert*-butyl groups (1.08 and 1.17 ppm) also confirms the existence of a plane of symmetry of resulting macrocycle **9** in the *cone* conformation. The signal in the ^77^Se NMR spectrum (95 MHz, CDCl_3_) shows a significant downfield shift (δ 321.4 ppm) compared to the starting macrocycle **8**.

The structure/conformation of **9** was unambiguously confirmed by single-crystal X-ray analysis (Figure 4). The macrocycle crystallized in the trigonal system, with space group *P*3_1_21, and adopts a pinched *cone* conformation. If we define the main plane of the molecule using the four bridging atoms, two opposite aromatic moieties are practically perpendicular to this plane (interplanar angles Φ = 88.11°), while the remaining two are tilted out of the cavity (Φ = 141.10°). Unfortunately, even in this case, it is not possible to separate the disordered positions of selenium and carbon bridges (50% probability) due to the presence of a 2-fold axis.

**Figure 4:**
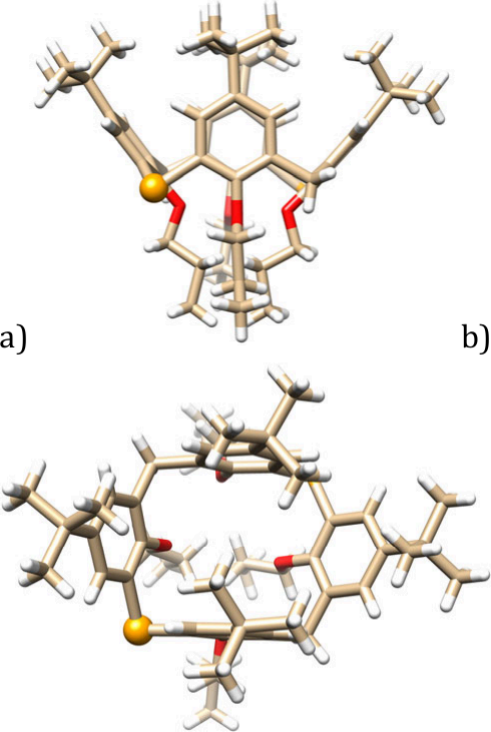
Single-crystal X-ray structures of compound **9**: (a) side view and (b) top view (one of the two equivalent positions of Se shown as a ball).

In conclusion, 2-selenacalix[4]­arene, a new member of the calixarene family with mixed bridges (−CH_2_– and −Se−), was prepared by two independent synthetic approaches. The basic dynamic behavior of the new system in solution was studied using VT NMR techniques. While the *cone*–*cone* equilibrium is only minimally affected by the introduced heteroatom, the change in the direction of the circular hydrogen bond (flip-flop motion) exhibits a lower value of Δ*G* ^#^, indicating weaker hydrogen bonds compared to the parent calix[4]­arene. As preliminary studies show, the selenium analogue of calix[4]­arene can be stereoselectively immobilized in the *cone* conformation, suggesting its potential use in host–guest chemistry, where the *cone* conformer is the most commonly used. On the other hand, the presence of the heteroatom offers a broader set of basic conformers (six different conformations) compared to the parent macrocycle; thus, further research will include studying the conformational preferences of alkylation reactions depending on the type of base, temperature, and/or solvent used. Moreover, as indicated by X-ray results, the Se bridge could also enable new types of complexes unknown in classical calixarenes (η^6^-chalcogen bonding). Further properties and applications of this system are currently being studied.

## Supplementary Material



## Data Availability

The data underlying this study are available in the published article and its Supporting Information.

## Notes

Experimental details, copies of ^1^H and ^13^C NMR and IR spectra and HRMS of new compounds, and X-ray crystallographic data for compounds (PDF)
