# Supplementary material for: Newcomer to the Calixarene Family: Synthesis and Characterization of Selenacalix[4]arene
Source: Org Lett. 2025 Dec 9;27(50):13722–7. doi: 10.1021/acs.orglett.5c04133 (PMC12723736; doi:10.1021/acs.orglett.5c04133)
Supplement: Supplementary file 1 [file ol5c04133_si_001.pdf]

Supporting Information

Newcomer to the Calixarene Family: Synthesis and Characterization of Selenacalix[4]arene

Michal Churý,[‡] Tadeáš Petrů,[‡] Radek Staník,[‡] Jan Sýkora,[†] Václav Eigner[§] and Pavel Lhoták[‡]*

[‡]Department of Organic Chemistry, University of Chemistry and Technology, Prague (UCTP), Technická 5, 166 28, Prague 6, Czech Republic.

[†]Department of Analytical Chemistry, UCTP, Technická 5, 166 28, Prague 6, Czech Republic.

[§]Institute of Physics AS CR, v.v.i., Na Slovance 2, 182 21, Prague 8, Czech Republic.

Table of Content

1. General information 2
2. Experimental procedures and characterization 2
3. Spectral characterization of compounds 13
4. Variable temperature NMR spectra 32
5. Crystallographic data 38
6. Single crystal X-ray structures 39

1. General information

All chemicals were purchased from commercial sources and used without further purification. Acetone was dried and distilled using conventional methods, THF, DMF and CH_3CN were dried using column solvent purification system PureSolv MD7 (Inert). Melting points were measured on Heiztisch Mikroskop Polytherm A (Wagner & Munz) and they are not corrected. ^1H, ^{13}C, ^{77}Se and VT spectra were measured on Agilent 400-MR DDR2 operating at 399.94 MHz for ^1H, JEOL JNM-ECZL400G operating at 399.38 MHz for ^1H and 100.54 MHz for ^{13}C, another JEOL JNM-ECZL400G operating at 399.38 MHz for ^1H and Bruker Avance^{III} 500 operating at 500.13 MHz for ^1H, 125.77 MHz for ^{13}C and 95.41 MHz for ^{77}Se. Chemical shifts are given in δ-units (ppm) and are referenced to solvent signal, ^{77}Se shifts are referenced to diphenylselenide. IR spectra were measured on FTIR spectrometer Nicolet iS50 ABX with diamond ATR module. The measurement parameters: spectral range 4000 – 400 cm^{-1}, resolution 4 cm^{-1}, 64 spectral accumulations. ESI HRMS spectra were measured on Q-TOF (Micromass) spectrometer. Substance purities and courses of the reactions were monitored by thin layer chromatography (TLC) using silica gel 60 F_{254} on aluminium-backed sheets (Merck) and analysed at 254 and 365 nm. Preparative TLC was carried out on self-prepared plates using silica gel 60 GF_{254} for thin-layer chromatography (Merck) on glass tables.

2. Experimental procedures and characterization

Compound 3, compound 6

All the starting compounds were prepared based on the previously published procedures.[S1, S2] Compound **6** was prepared from compound **7**, listed below.

2,2'-Methylenebis(4-*tert*-butylphenol) (7)

The synthesis of this compound was carried out by a modified literature procedure[S3] from 6,6'-methylenebis-(2,4-di-*tert*-butylphenol).

6,6'-Methylenebis-(2,4-di-*tert*-butylphenol)[S3] (109 g, 0,257 mol) was dissolved in 1.3 l of toluene and then cooled down in an ice bath. In another flask, 68.4 g of $AlCl_3$ was dissolved in 153 ml of nitromethane and then diluted with another 875 ml of toluene. The $AlCl_3$ solution was then added to the 6,6'-methylenebis-(2,4-di-tert-butylphenol) solution and stirred thoroughly for 2 hours. After the reaction was complete the crude reaction mixture was carefully quenched by 1 l of 1M HCl. The aqueous phase was separated and extracted two times with 250 ml of diethyl ether. All the organic material was mixed and dried over $MgSO_4$. After evaporation of the solvents, the mixture was triturated with hexanes, affording 56.90 g (70%) of fine white powder. Analytical data were identical to those previously reported. [S3]

2,2'-Selenobis(4-*tert*-butylphenol) (2)

First, a solution of $SeCl_2$ was prepared. To 2.10 g (26.6 mmol) of elemental selenium, 2.2 ml (3.7 g, 27 mmol) of SO_2Cl_2 was added. The mixture was stirred at room temperature for 10 minutes, then 30 ml of dry tetrahydrofurane was added and the mixture was stirred for 1 h. To this mixture, 8.00 g (53.3 mmol) of 4-*tert*-butylphenol in 60 ml of dry THF was added and the mixture was left to stir overnight. The crude mixture was then evaporated *in vacuo*, diluted with dichloromethane, filtered and separated using column chromatography (silica gel, eluent = dichlormethane:cyclohexane 4:1). The product was isolated in 37% yield (3,70 g) as a brownish oil.

^1H NMR ($CDCl_3$, 400 MHz, 298 K) δ (ppm): 7.41 (d, J = 2.3 Hz, 2H, Ar-H), 7.25 (dd, J = 8.4, 2.6 Hz, 2H, Ar-H), 6.88 (d, J = 8.5 Hz, 2H, Ar-H), 6.04 (bs, 2H, Ar-OH), 1.23 (s, 18H, -CH_3).

$^{13}C\{^1H\}$ NMR ($CDCl_3$, 126 MHz, 298 K) δ (ppm): 153.2, 144.6, 132.2, 127.8, 115.2, 114.8, 34.2, 31.4.
^{77}Se NMR ($CDCl_3$, 95 MHz, 298 K) δ (ppm): 191.56.
IR (ATR) ν (cm^{-1}): 3401, 3069, 2958, 2905, 2867, 1700, 1597, 1582, 1483, 1397, 1364, 1335.
HRMS (ESI) *m/z*: $[M - H]^-$ Calcd for $C_{20}H_{25}O_2Se$ 377.1025; Found 377.1024.

3,3'-Selenobis(5-*tert*-butyl-2-hydroxybenzaldehyde) (4)

Benzaldehyde **3 (**10,40 g, 58.3 mmol), SeO_2 (3.90 g, 35.1 mmol) and 60 ml of dry pyridine were mixed together and heated using a heating mantle to 110 °C for 7 days. The reaction was quenched by adding 5M HCl until the pH of 2-3 was achieved. The mixture was then extracted 4 times with dichloromethane, washed with 1M HCl and with brine, and dried over $MgSO_4$. Column chromatography of this mixture yielded 6.90 g (54 %) of yellow powder.
M.p. 115 – 119 °C.
^1H NMR ($CDCl_3$, 500 MHz, 298 K) δ (ppm): 11.33 (s, 2H, Ar-O*H*), 9.90 (s, 2H, -C*H*=O), 7.57 (d, *J* = 2.4 Hz, 2H, Ar-*H*), 7.51 (d, *J* = 2.4 Hz, 2H, Ar-*H*), 1.23 (s, 18H, -CH_3).
$^{13}C\{^1H\}$ NMR ($CDCl_3$, 126 MHz, 298 K) δ (ppm): 196.4, 158.3, 143.7, 139.0, 129.9, 119.9, 117.9, 34.2, 31.2.
^{77}Se NMR ($CDCl_3$, 95 MHz, 298 K) δ (ppm): 291.36.
IR (ATR) ν (cm^{-1}): 3169, 3115, 3062, 3035, 2957, 2923, 2866, 1646, 1609, 1447, 1410, 1357.
HRMS (ESI) *m/z*: $[M + Na]^+$ Calcd for $C_{22}H_{26}O_4SeNa$ 457.0889; Found 457.0891; $[M - H]^-$ Calcd for $C_{22}H_{25}O_4Se$ 433.0924; Found 433.0921.

6,6'-Selenobis(4-*tert*-butyl-2-hydroxymethylphenol) (5)

To a solution of **4** (437 mg, 1 mmol) in 30 ml of dry THF, 77 mg of $NaBH_4$ (2 mmol) were added. The mixture was let to stir for 2 h, then 1M HCl was added to quench the reaction. The mixture was extracted twice with diethyl ether, once with water and once with brine, and dried over $MgSO_4$. After evaporation of the solvents the product was obtained as a yellowish sticky mass (quantitative yield), containing small amount of THF.
^1H NMR ($CDCl_3$, 400 MHz, 298 K) δ (ppm): 7.76 (bs, 2H, Ar-O*H*), 7.32 (d, *J* = 2.3 Hz, 2H, Ar-*H*), 7.10 (d, *J* = 2.3 Hz, 2H, Ar-*H*), 4.76 (s, 4H, -CH_2-), 2.88 (bs, 2H, -O*H*), 1.21 (s, 18H, -CH_3).
$^{13}C\{^1H\}$ NMR ($CDCl_3$, 126 MHz, 298 K) δ (ppm): 152.5, 143.9, 131.6, 126.2, 125.1, 116.2, 64.0, 34.2, 31.4.
^{77}Se NMR ($CDCl_3$, 95 MHz, 298 K) δ (ppm): 204.16.
IR (ATR) ν (cm^{-1}): 3358, 2958, 2908, 2867, 1703, 1656, 1603, 1579, 1479, 1464, 1429, 1394, 1362.
HRMS (ESI) *m/z*: $[M + Na]^+$ Calcd for $C_{22}H_{30}O_4SeNa$ 461.1202; Found 461.1201; $[M - H]^-$ Calcd for $C_{22}H_{29}O_4Se$ 437.1236; Found 437.1236.

Cyclisation reactions
General procedure 1
p-Toluenesulfonic acid monohydrate (380 mg, 2 mmol) was suspended in 85 ml of dry $CHCl_3$ and the mixture was brought using a heating mantle to reflux. To the boiling mixture, two solutions of corresponding dimers (1 mmol in 17 ml of $CHCl_3$ each) were simultaneously added using a syringe pump during 4 h. The mixture was then refluxed for another hour, followed by addition of 100 ml of water. After mixing thoroughly, the two layers were separated, the organic layer was further extracted with water and then dried using $MgSO_4$. The drying agent was filtered off, the solution was evaporated *in vacuo* and triturated with 15 ml of methanol. The white precipitate was separated using a centrifuge, the trituration-centrifugation step was then repeated twice. The resulting powder was then dried in the air.

Reaction of 2 and 6
For the reaction, 376 mg (1.00 mmol) of **2**, 372 mg (1.00 mmol) of **6** and 380 mg (2.00 mmol) of *p*-toluenesulfonic acid monohydrate were used. The reaction yielded 419 mg (59 %) of product as a white powder. The powder contained compound **8** with some minor impurities.

Reaction of 5 and 7
For the reaction, 441 mg (1.01 mmol) of **5**, 315 mg (1.01 mmol) of **7** and 383 mg (2.01 mmol) of *p*-toluenesulfonic acid monohydrate were used. The reaction yielded 478 mg (66 %) of white powder. The powder contained compound **8** with some minor impurities.

5,11,17,23-Tetra-*tert*-butyl-2-selena-calix[4]arene (8)
For further reactions, the product was not further purified. For analyses, the product was recrystallized from boiling acetone.
M.p. >300 °C.
^{1}H NMR ($CDCl_3$, 400 MHz, 298 K) δ (ppm): 9.98 (s, 4H, -O*H*), 7.55 (d, *J*=2.5 Hz, 2H, Ar-*H*), 7.23 (d, *J*=2.5 Hz, 2H, Ar-*H*), 7.08 (d, *J* = 2.4 Hz, 2H, Ar-*H*), 7.06 (d, *J* = 2.4 Hz, 2H, Ar-*H*), 4.25 (bs, 3H, Ar-CH_2-Ar), 3.53 (bs, 3H, Ar-CH_2-Ar), 1.22 (s, 18H, -CH_3), 1.21 (s, 18H, -CH_3).
^{13}C{^{1}H} NMR ($CDCl_3$, 126 MHz, 298 K) δ (ppm): 150.7, 146.6, 144.6, 144.5, 133.8, 129.8, 127.8, 127.2, 127.1, 126.0, 125.7, 117.3, 34.1, 34.0, 33.0, 32.5, 31.40, 31.38.
^{77}Se NMR ($CDCl_3$, 95 MHz, 298 K) δ (ppm): 230.13.
IR (ATR) ν (cm^{-1}): 3185, 3056, 3022, 2956, 2905, 2868, 1752, 1715, 1605, 1579, 1482, 1456, 1423, 1394, 1362.
HRMS (ESI) *m/z*: [M - H]$^-$ Calcd for $C_{43}H_{53}O_4Se$ 713.3115; Found 713.3135.

General procedure 2

p-Toluenesulfonic acid monohydrate (760 mg, 4 mmol) was suspended in 175 ml of dry toluene and the mixture was heated using a heating mantle to 100°C. The two solutions of corresponding dimers (2 mmol in 35 ml of toluene each) were simultaneously added to this mixture, using a syringe pump during 16 h. The mixture was then heated for another 6 h, followed by addition of 100 ml of water. After mixing thoroughly, the two layers were separated and the organic layer was further extracted twice with water and then evaporated.

Reaction of 2 and 6

For the reaction, 756 mg (2.00 mmol) of **2**, 746 mg (2.00 mmol) of **6** and 770 mg (4.05 mmol) of *p*-toluenesulfonic acid monohydrate were used. The dimer **6** was added in a mixture of 28 ml of toluene and 7 ml of THF instead of pure toluene due to low solubility. After evaporation, the mixture was triturated with acetone and centrifuged. This process was repeated three times, yielding 126 mg (8.8 %) of white powder **8**. The residue was further analysed by HRMS.

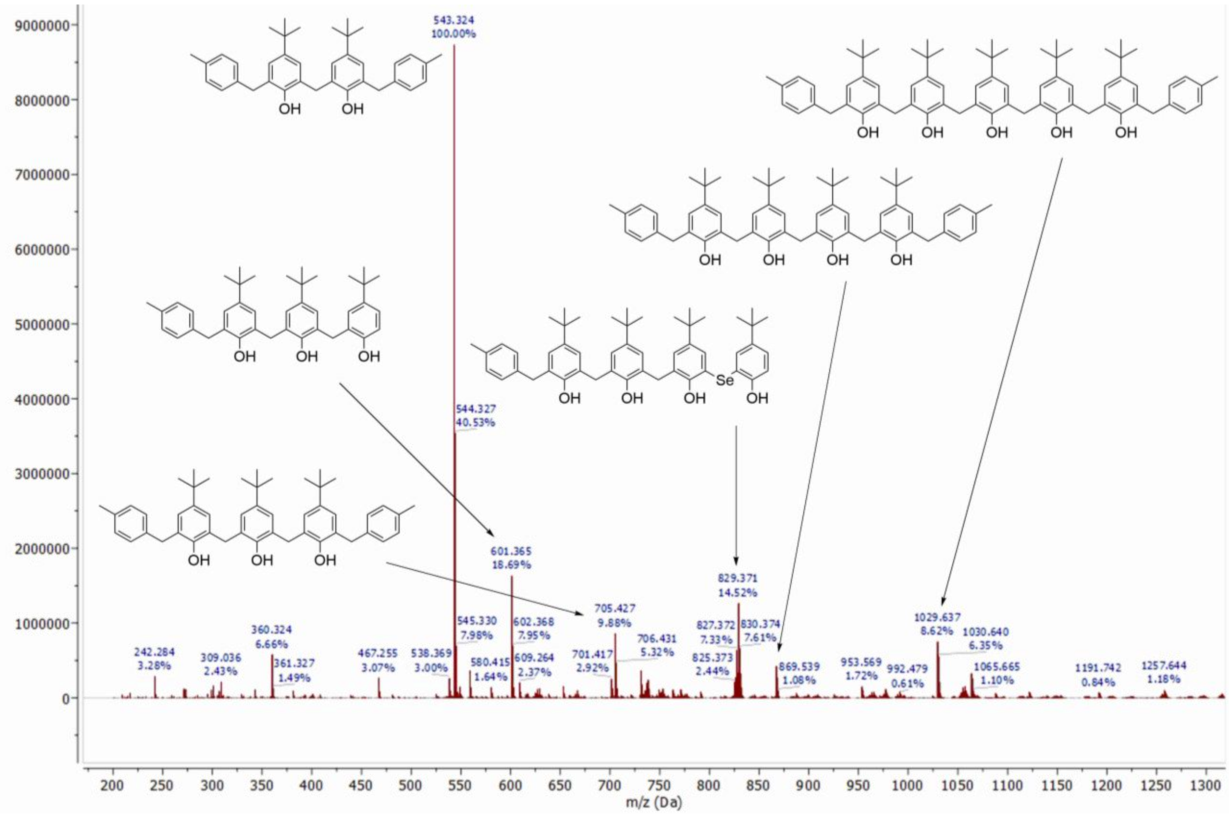

Figure S1: HRMS spectrum of the residue of the reaction of **2** and **6** according to General procedure 2 in positive mode

Table S1: Predicted byproducts found in HRMS spectrum (positive mode) of the residue from the reaction of **2** and **6** according to General procedure 2.

Structure	*m/z* calc	*m/z* found
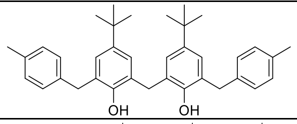	543.3234 [M +Na]$^+$	543.3239 [M +Na]$^+$
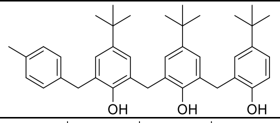	601.3652 [M +Na]$^+$	601.3650 [M +Na]$^+$
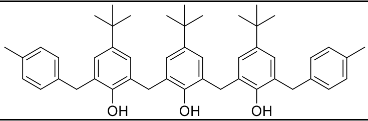	705.4278 [M +Na]$^+$	705.4274 [M +Na]$^+$
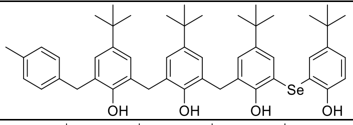	829.3706 [M +Na]$^+$	829.3707 [M +Na]$^+$
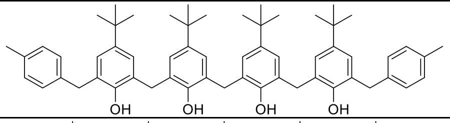	867.5323 [M +Na]$^+$	867.5316 [M +Na]$^+$
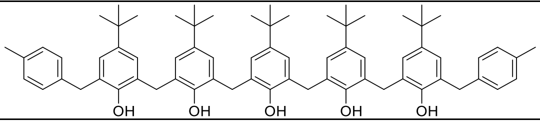	1029.6368 [M +Na]$^+$	1029.6367 [M +Na]$^+$

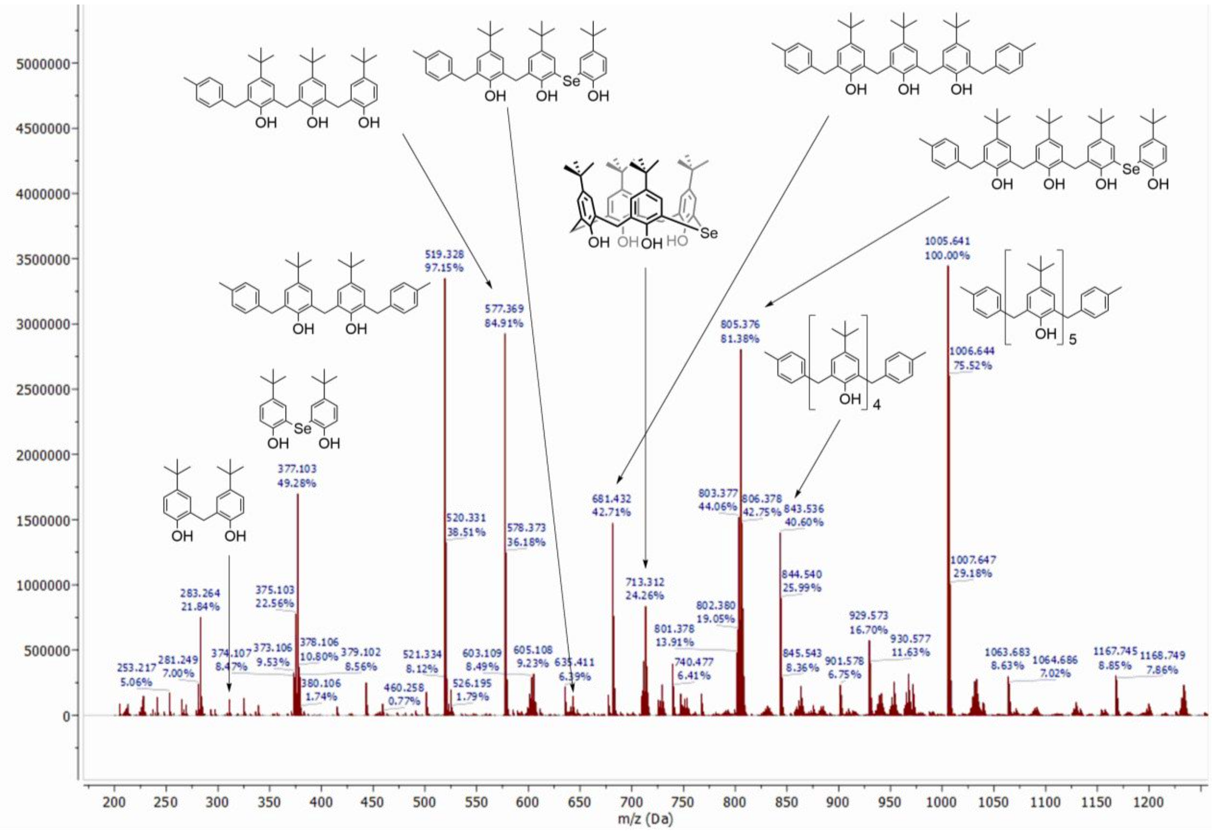

Figure S2: HRMS spectrum (negative mode) of the crude reaction mixture (reaction of **2** and **6)** according to General procedure 2. The residual **8** is also shown.

Table S2: Predicted byproducts found in HRMS spectrum (negative mode) of crude reaction mixture (reaction of **2** and **6**) according to General procedure 2.

Structure	*m/z* calc	*m/z* found
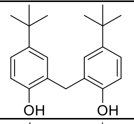	311.2017 $[M - H]^-$	311.2017 $[M - H]^-$
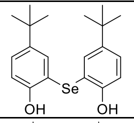	377.1025 $[M - H]^-$	377.1025 $[M - H]^-$
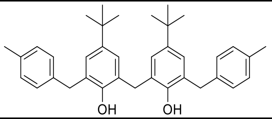	519.3269 $[M - H]^-$	519.3276 $[M - H]^-$
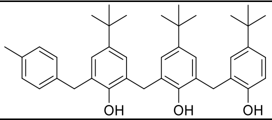	577.3687 $[M - H]^-$	577.3693 $[M - H]^-$
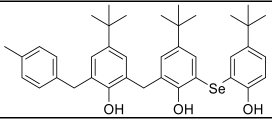	643.2696 $[M - H]^-$	643.2700 $[M - H]^-$
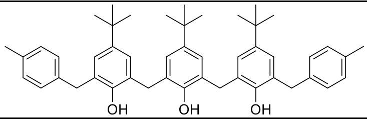	681.4313 $[M - H]^-$	681.4320 $[M - H]^-$
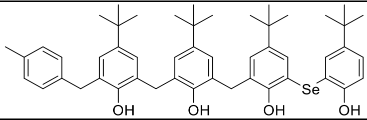	805.37406 $[M - H]^-$	805.37555 $[M - H]^-$
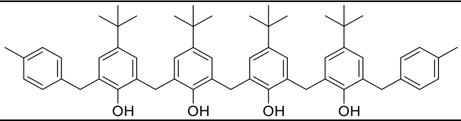	843.53578 $[M - H]^-$	843.53601$[M - H]^-$
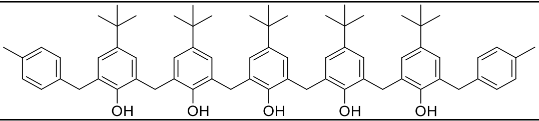	1005.64025 $[M - H]^-$	1005.64093 $[M - H]^-$

Reaction of 5 and 7

For the reaction, 877 mg of **5** (2.01 mmol), 626 mg of **7** (2.00 mmol) and 765 mg (4.02 mmol) of *p*-toluenesulfonic acid monohydrate were used. The reaction mixture was analysed using ^{1}H NMR and mass spectrometry and product **8** was not found. In mass spectra, however, a lot of possible byproducts and unreacted **7** could be found. The starting dimer **5** converted completely.

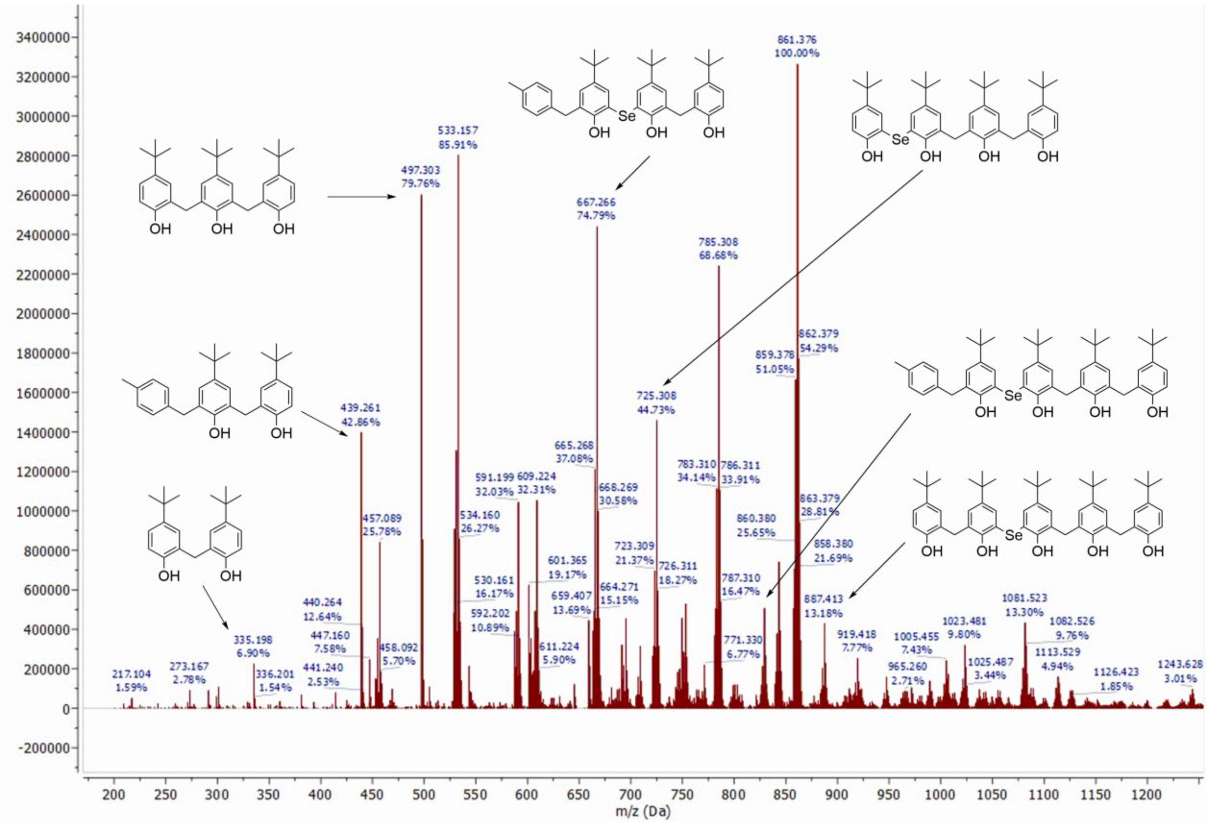

Figure S3: HRMS spectrum (positive mode) of reaction of **5** and **7** according to General procedure 2.

Table S3: Predicted byproducts found in HRMS spectrum (positive mode) of the reaction mixture of **5** and **7** according to General procedure 2.

Structure	*m/z* calc	*m/z* found
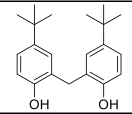	335.1982 [M +Na]$^{+}$	335.1981 [M +Na]$^{+}$
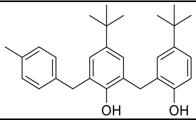	439.2608 [M +Na]$^{+}$	439.2608 [M +Na]$^{+}$
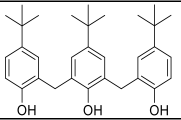	497.3026 [M +Na]$^{+}$	497.3026 [M +Na]$^{+}$
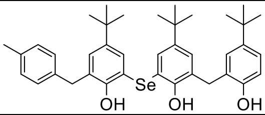	667.2661 [M +Na]$^{+}$	667.2663 [M +Na]$^{+}$
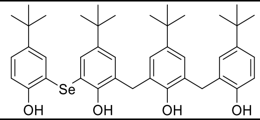	725.3080 [M +Na]$^{+}$	725.3079 [M +Na]$^{+}$
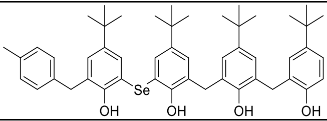	829.3706 [M +Na]$^{+}$	829.3717 [M +Na]$^{+}$
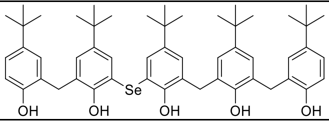	887.4124 [M +Na]$^{+}$	887.4130 [M +Na]$^{+}$

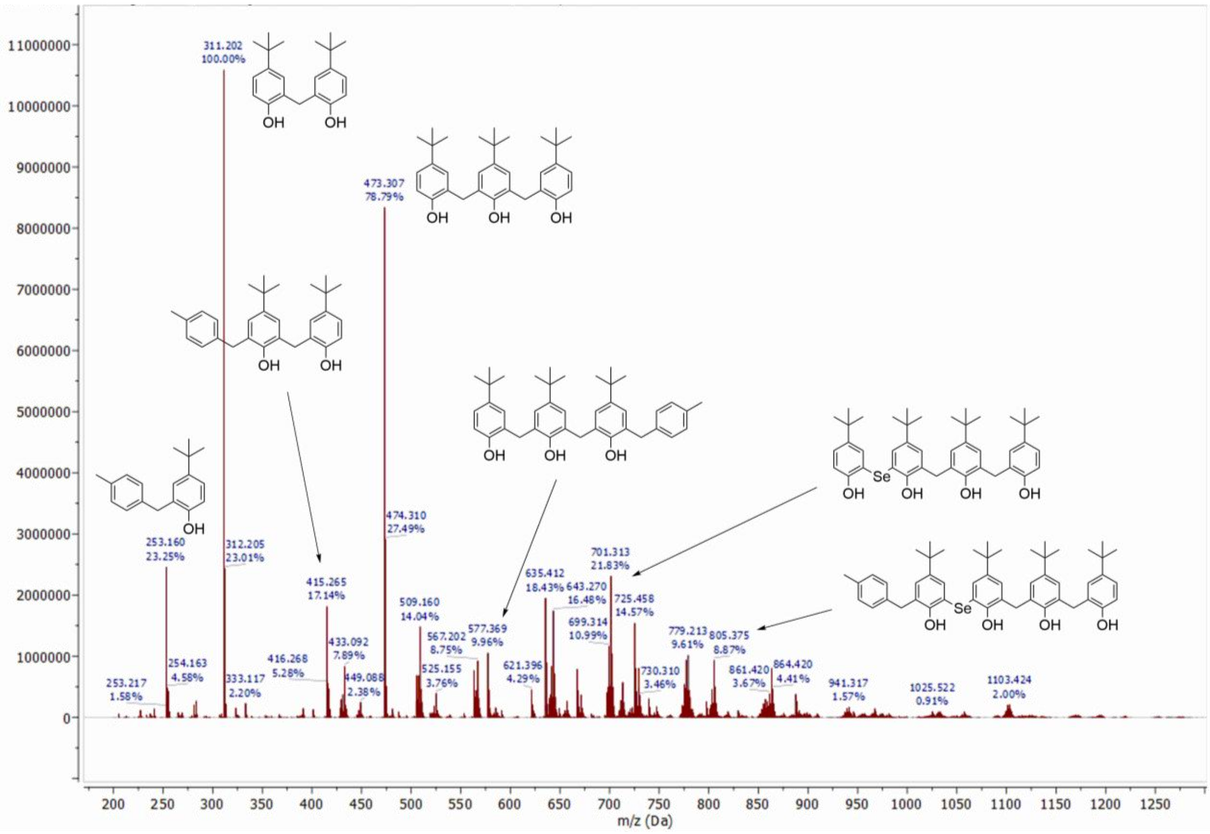

Figure S4: HRMS spectrum (negative mode) of reaction of **5** and **7** according to General procedure 2.

Table S4: Predicted byproducts found in HRMS spectrum (negative mode) of the reaction mixture of **5** and **7** according to General procedure 2.

Structure	*m/z* calc	*m/z* found
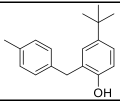	253.1598 [M - H]$^-$	253.1599 [M - H]$^-$
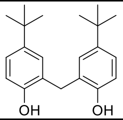	311.2017 [M - H]$^-$	311.2020 [M - H]$^-$
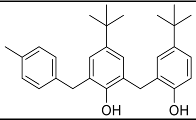	415.2643 [M - H]$^-$	415.2647 [M - H]$^-$
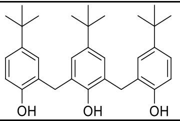	473.3061 [M - H]$^-$	473.3066 [M - H]$^-$
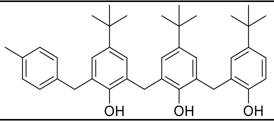	577.3687 [M - H]$^-$	577.3692 [M - H]$^-$
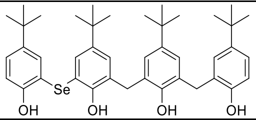	701.3115 [M - H]$^-$	701.3126 [M - H]$^-$
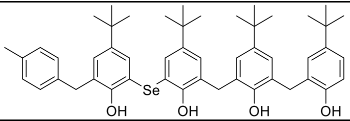	805.3741 [M - H]$^-$	805.3752 [M - H]$^-$

5,11,17,23-Tetra-*tert*-butyl-25,26,27,28-tetrapropoxy-2-selena-calix[4]arene (*Cone*) (9)

A solution of **8** (50 mg, 0.070 mmol) in 2 ml of dry DMF was cooled down to 0 °C and then 50,5 mg of 60% dispersion of NaH (1.26 mmol, 18 eq.) in mineral oil was added. The mixture was stirred for 30 min and then 164 µl of PrI (287 mg, 1.69 mmol, 24 eq.) was added. The reaction was then stirred for further 5 days at room temperature. The reaction was quenched by dropwise addition of 3 ml of 1M HCl. The mixture was then diluted with water and extracted four times with dichloromethane. Mixed extracts were washed with brine and dried over $MgSO_4$. After evaporation of the solvent, the product was isolated using preparative TLC (silica gel, eluent dichloromethane:cyclohexane 1:2), as a white solid, 30 mg (49 %).

M.p. 212-224 °C

^{1}H NMR ($CDCl_3$, 500 MHz, 298 K) δ (ppm): 7.28 (d, *J*=2.4 Hz, 2H, Ar-*H*), 6.84 (d, *J*=2.3 Hz, 2H, Ar-H), 6.83 (d, *J*=2.4 Hz, 2H, Ar-*H*), 6.81 (d, *J*=2.3 Hz, 2H, Ar-*H*), 4.46 (d, *J* = 12.7 Hz, 2H, Ar-CH_2-Ar), 4.42 (d, *J* = 12.5 Hz, 1H, Ar-CH_2-Ar), 4.03 (td, *J* = 9.2, 5.8 Hz, 2H, -O-CH_2-), 3.84 (td, *J* = 9.2, 5.8 Hz, 2H, -O-CH_2-), 3.84 – 3.73 (m, 4H, -O-CH_2-), 3.14 (d, *J* = 12.5 Hz, 1H, Ar-CH_2-Ar), 3.11 (d, *J* = 12.7 Hz, 2H, Ar-CH_2-Ar), 2.15 – 1.85 (m, 8H, -CH_2-), 1.12 (s, 18H, -CH_3), 1.03 (s, 18H, -CH_3), 1.03 (t, *J*=7.5 Hz, 6H, -CH_3), 0.99 (t, *J*=7.5 Hz, 6H, -CH_3).

^{13}C{^{1}H} NMR ($CDCl_3$, 101 MHz, 298 K) δ (ppm): 156.7, 153.9, 145.1, 144.4, 134.4, 134.2, 133.6, 132.9, 127.9, 125.5, 125.3, 124.9, 77.8, 77.1, 34.0, 34.0, 31.6, 31.5, 31.2, 31.1, 23.5, 23.4, 10.6, 10.4.

^{77}Se NMR ($CDCl_3$, 95 MHz, 298 K) δ (ppm): 321.42.

IR (ATR) ν (cm^{-1}): 2956, 2933, 2901, 2873, 1715, 1598, 1481, 1456, 1387, 1361.

HRMS (ESI) *m/z*: $[M + Na]^+$ Calcd for $C_{55}H_{78}O_4SeNa$ 905.4958; Found 905.4964.

(S1) Hofslokken, N. U.; Skattebol, L., Convenient Method for the *ortho*-Formylation of Phenols. *Acta Chem. Scand.* **1999,** *53* (4), 258-262.

(S2) Kim, S. Y.; No, K., Synthesis of Calixsalen: A Route to Azacalixarene Analogue. *Bull. Korean Chem. Soc.* **2007,** *28* (2), 315-318.

(S3) Ballmann, J.; Fuchs, M. G. G.; Dechert, S.; John, M.; Meyer, F., Synthesis and Coordination Properties of Chelating Dithiophenolate Ligands. *Inorg. Chem.* **2009,** *48* (1), 90-99.

3. Spectral characterization of compounds

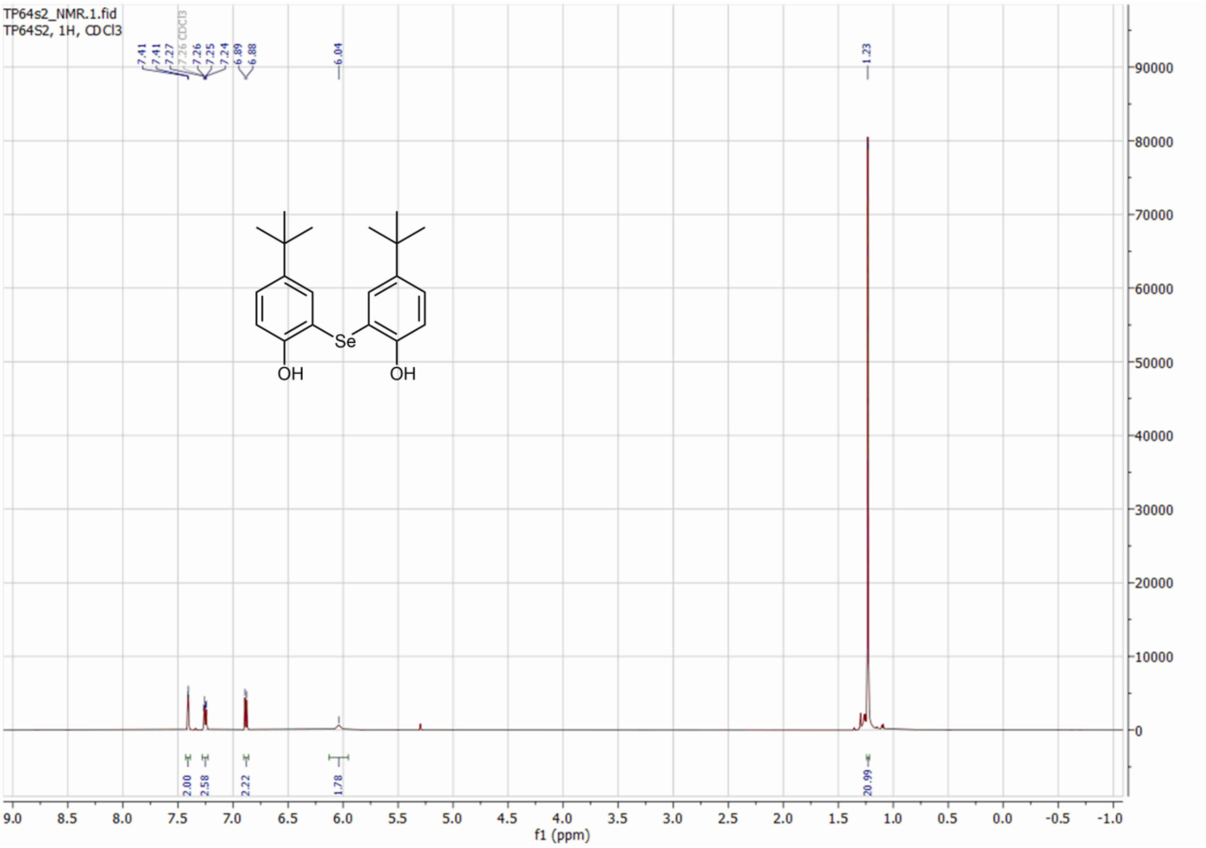

Figure S5: ^{1}H NMR spectrum of compound **2** in $CDCl_3$

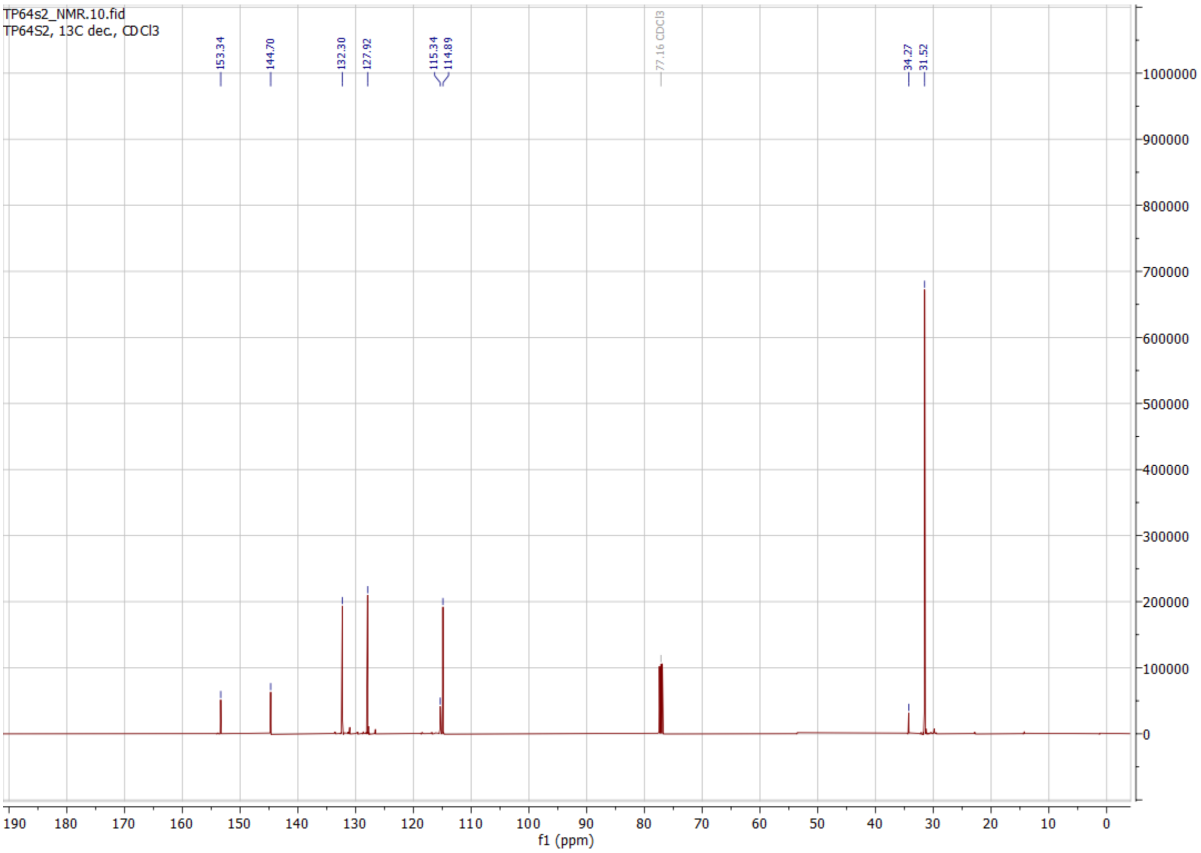

Figure S6: $^{13}C\{^{1}H\}$ NMR spectrum of compound **2** in $CDCl_3$

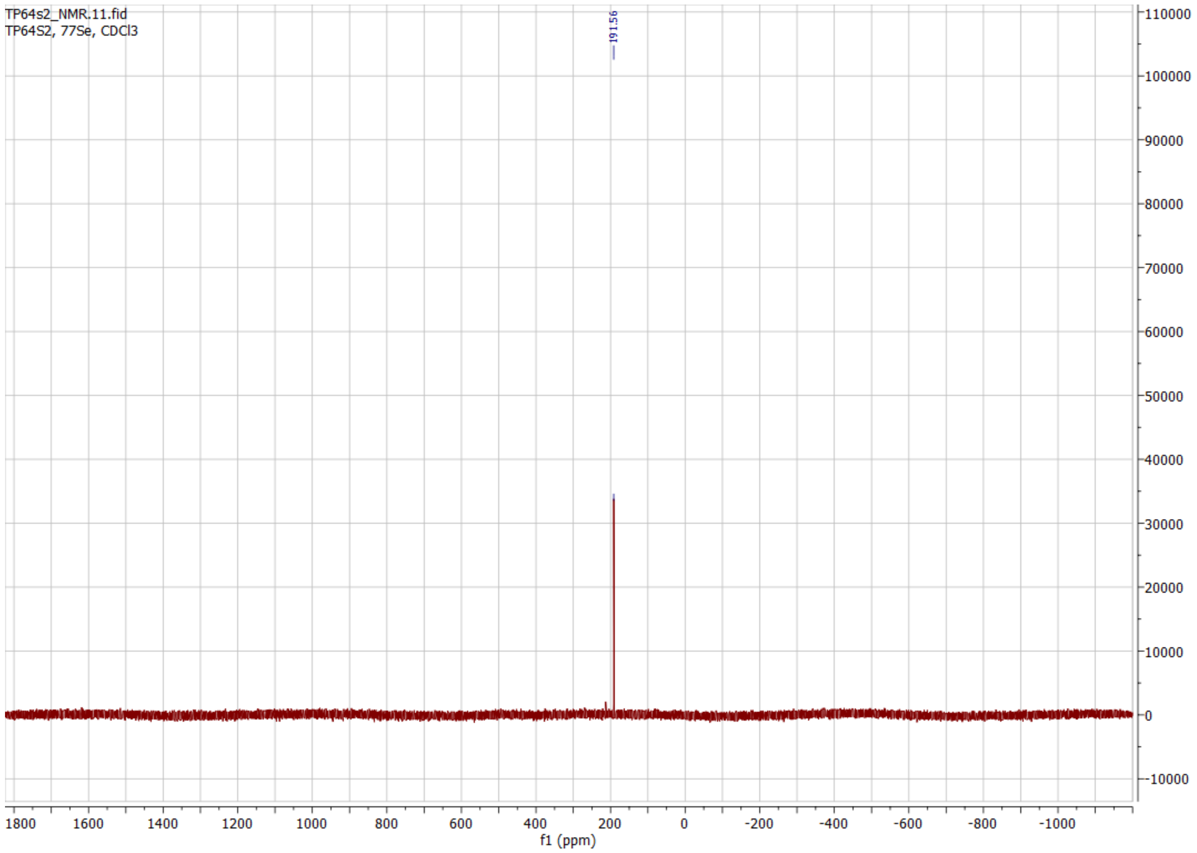

Figure S7: ^{77}Se NMR spectrum of compound **2** in $CDCl_3$

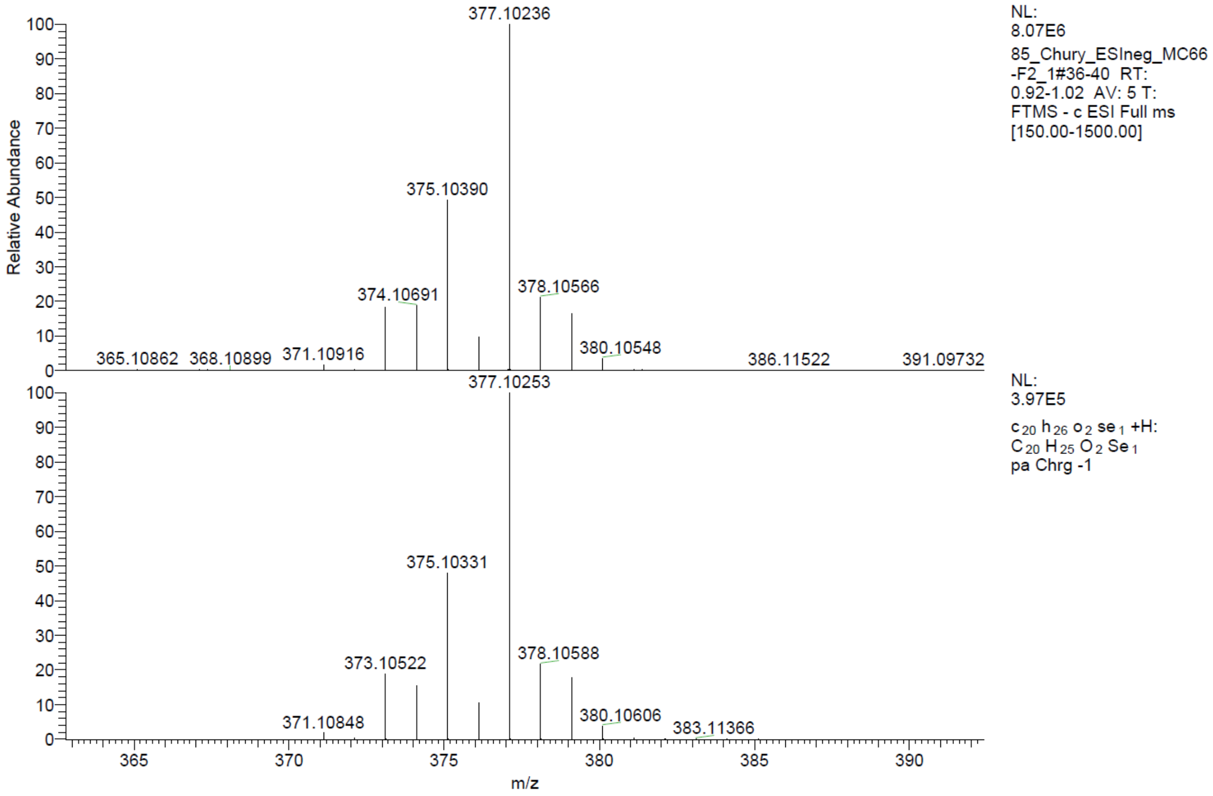

Figure S8: HRMS spectrum of compound **2**

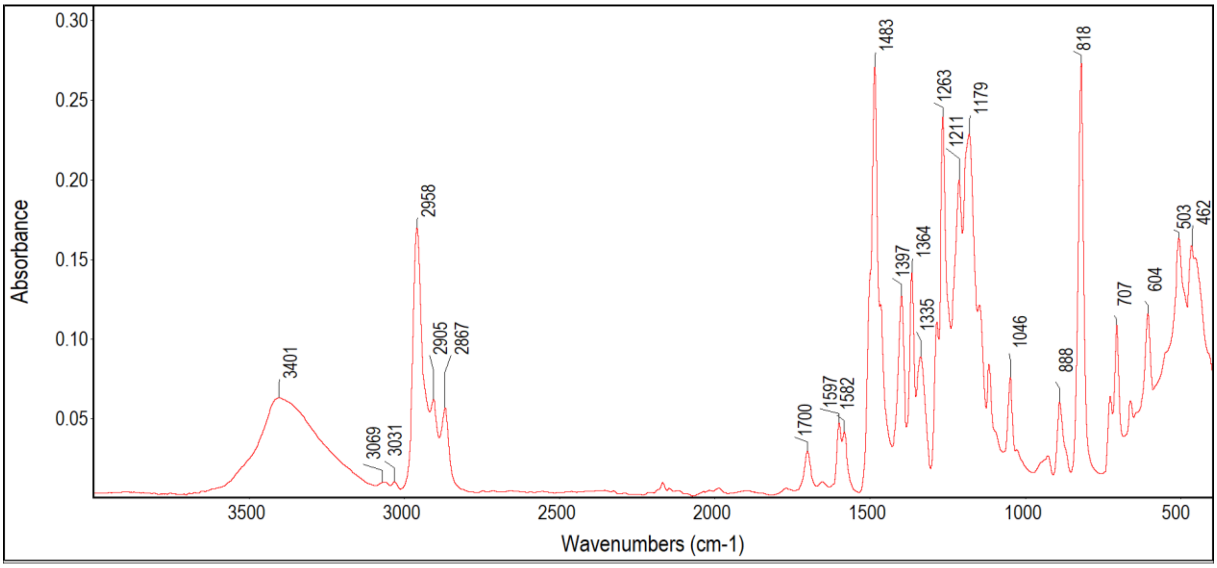

Figure S9: IR spectrum of compound **2**

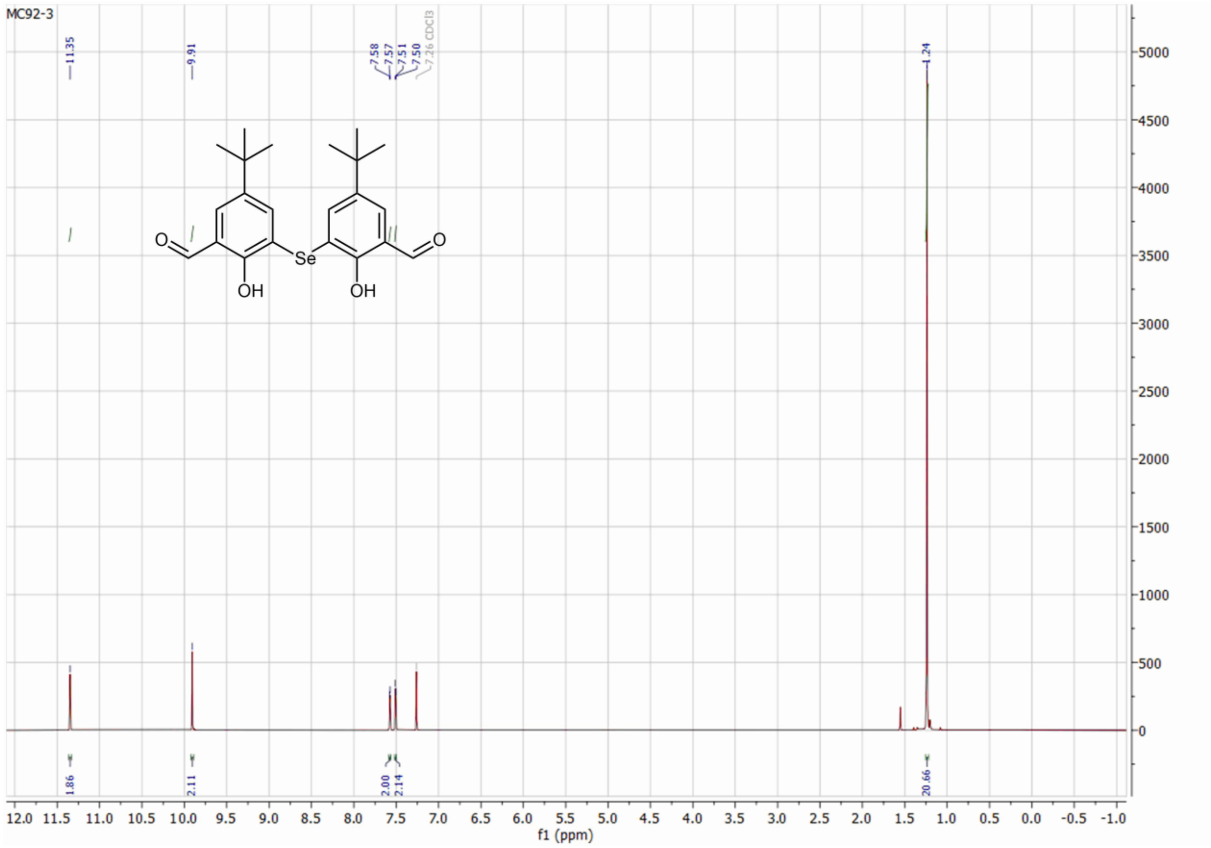

Figure S10: ^{1}H NMR spectrum of compound **4** in $CDCl_3$

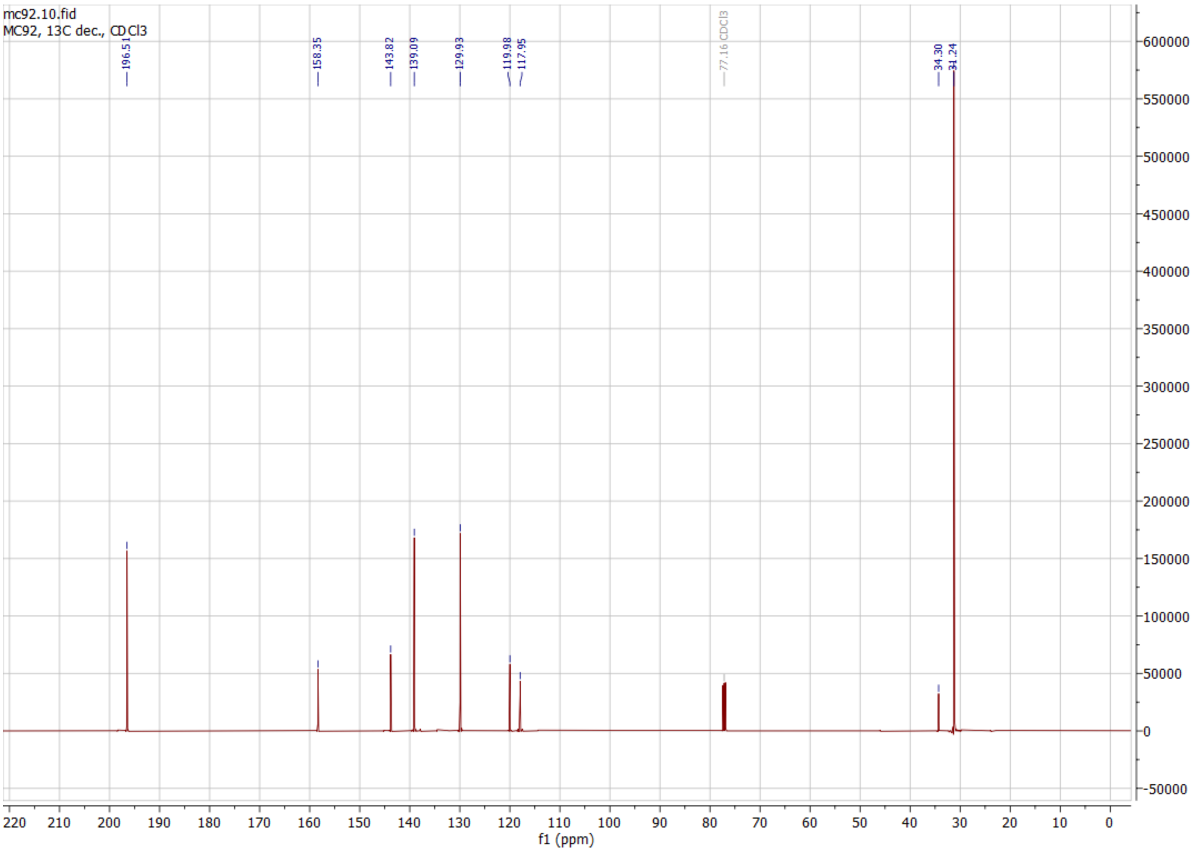

Figure S11: $^{13}C\{^{1}H\}$ NMR spectrum of compound **4** in $CDCl_3$

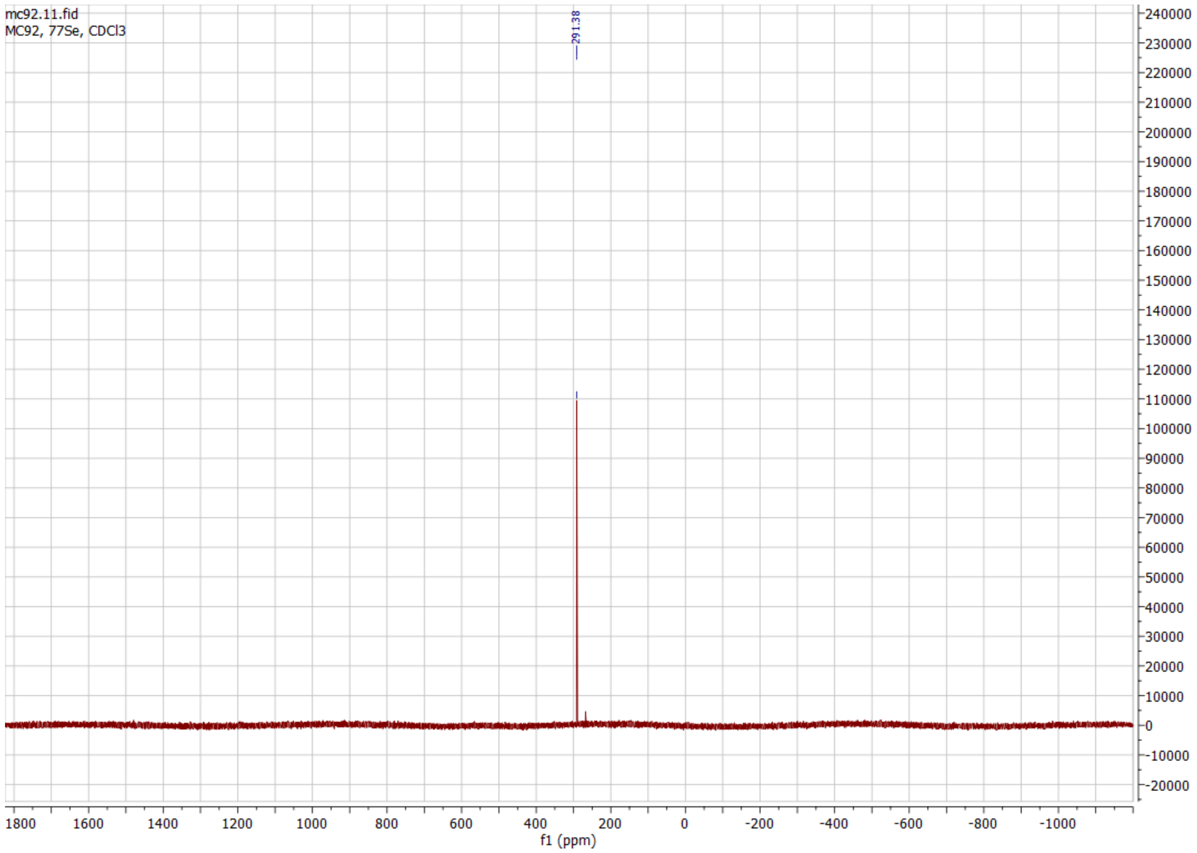

Figure S12: ^{77}Se NMR spectrum of compound **4** in $CDCl_3$

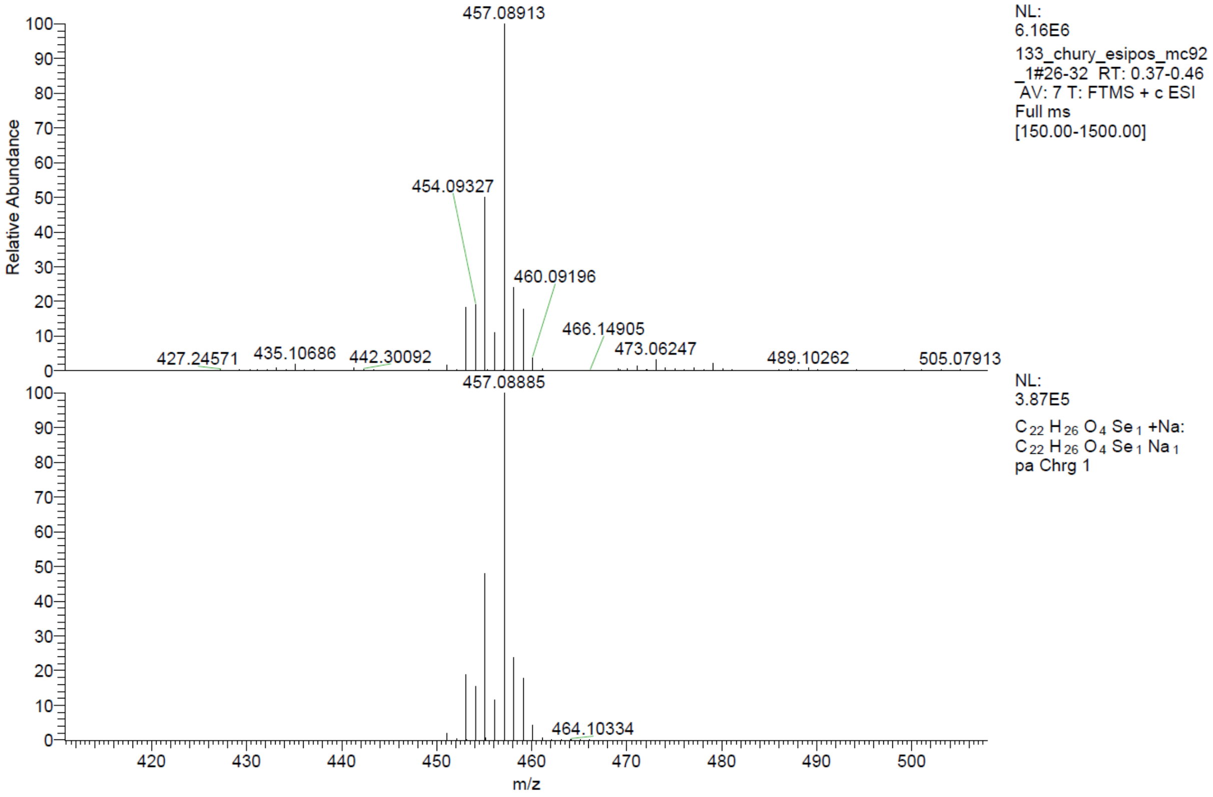

Figure S13: HRMS spectrum of compound **4** in positive mode

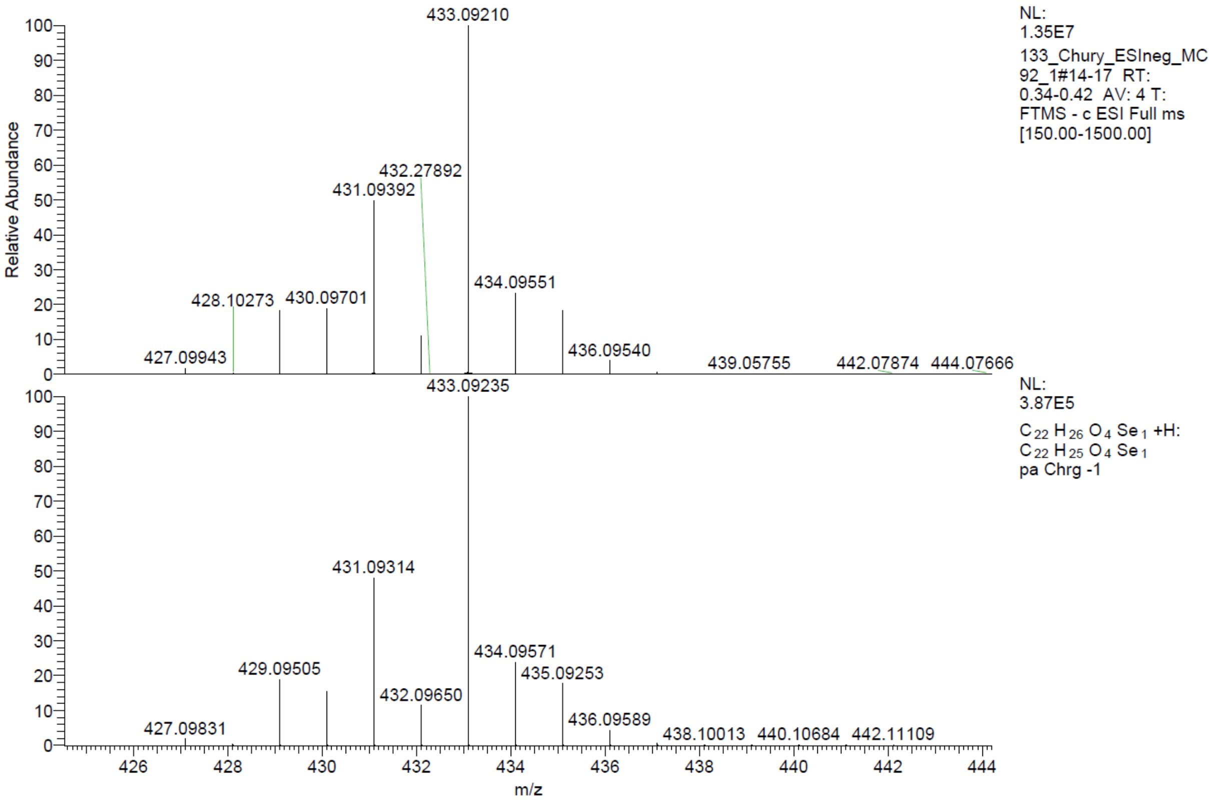

Figure S14: HRMS spectrum of compound **4** in negative mode

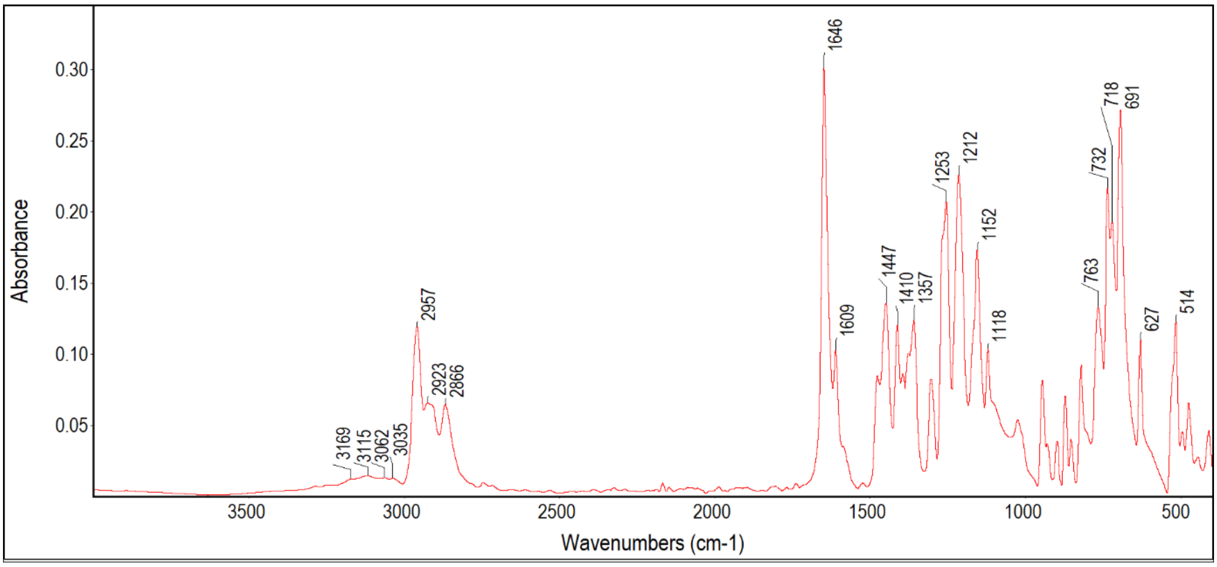

Figure S15: IR spectrum of compound **4**

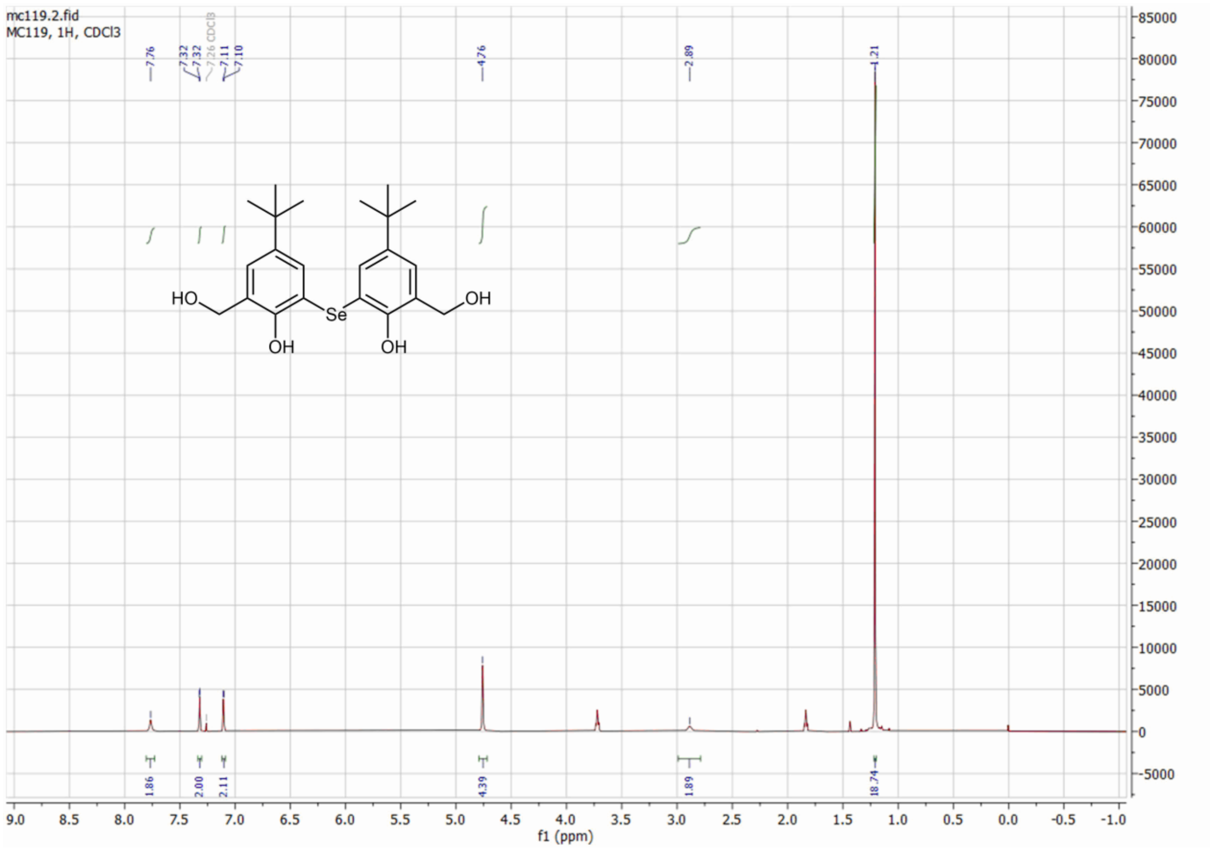

Figure S16: ^{1}H NMR spectrum of compound **5** in $CDCl_3$. Residual peaks of THF can be seen.

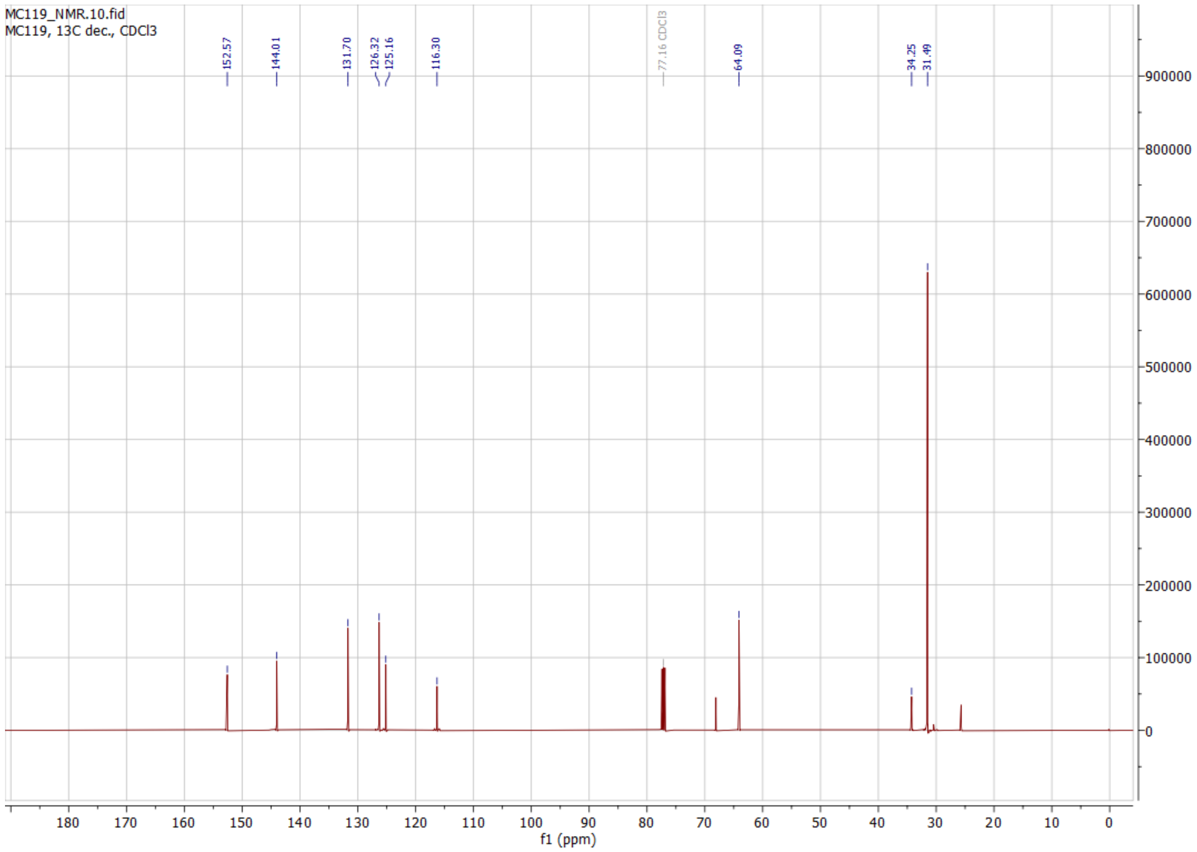

Figure S17: $^{13}C\{^{1}H\}$ NMR spectrum of compound **5** in $CDCl_3$

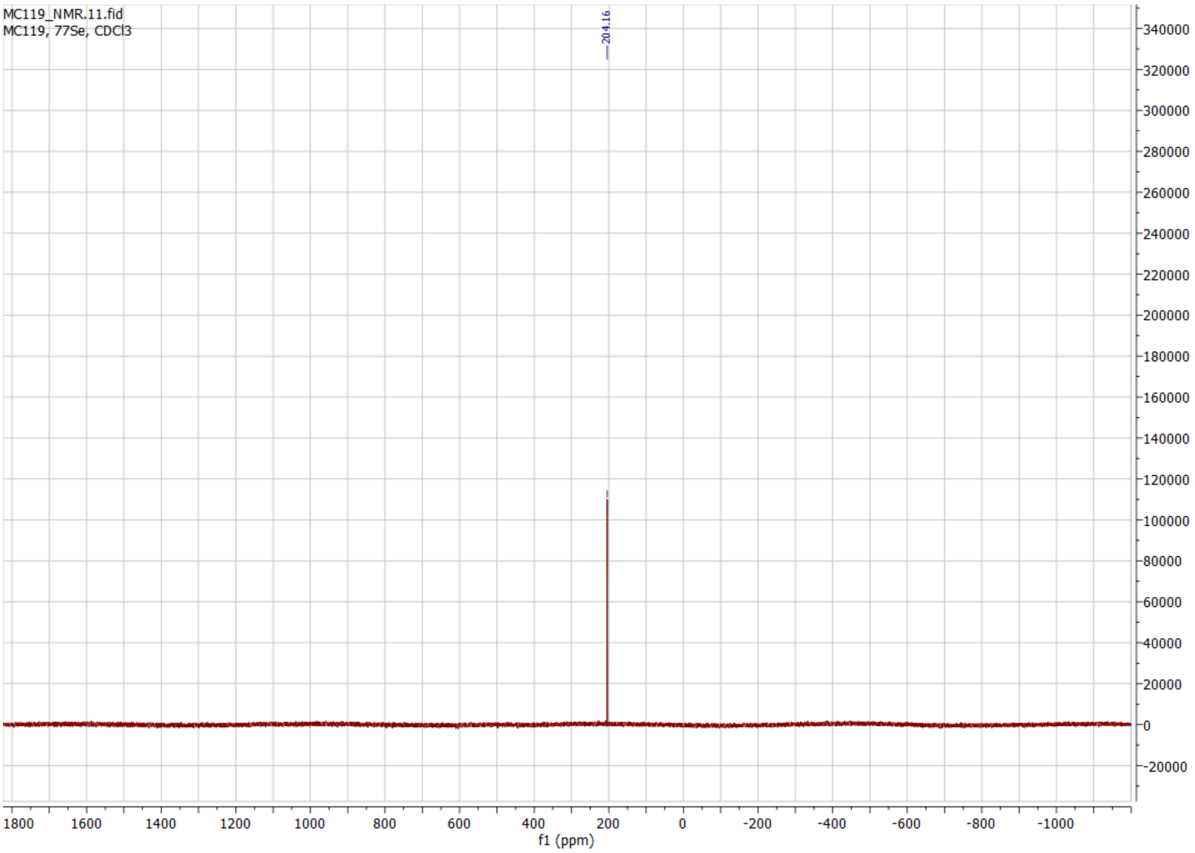

Figure S18: ^{77}Se NMR spectrum of compound **5** in $CDCl_3$

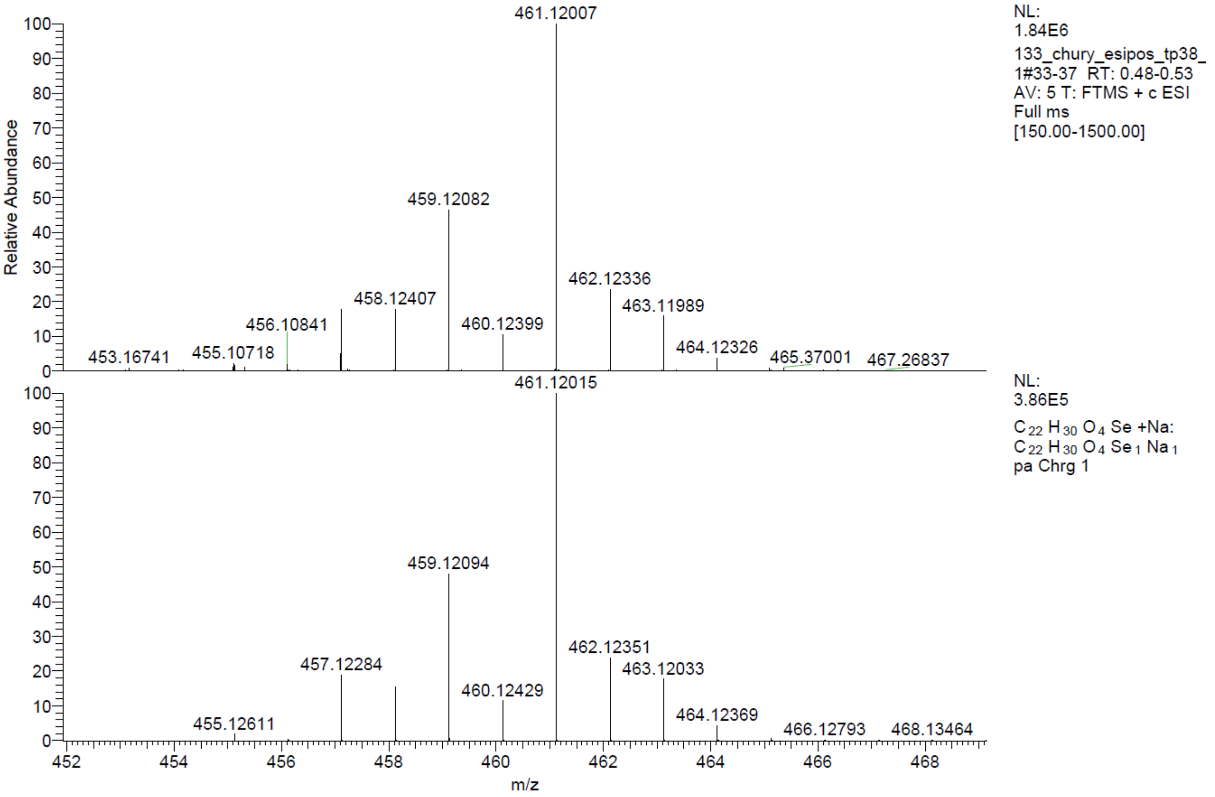

Figure S19: HRMS spectrum of compound **5** in positive mode

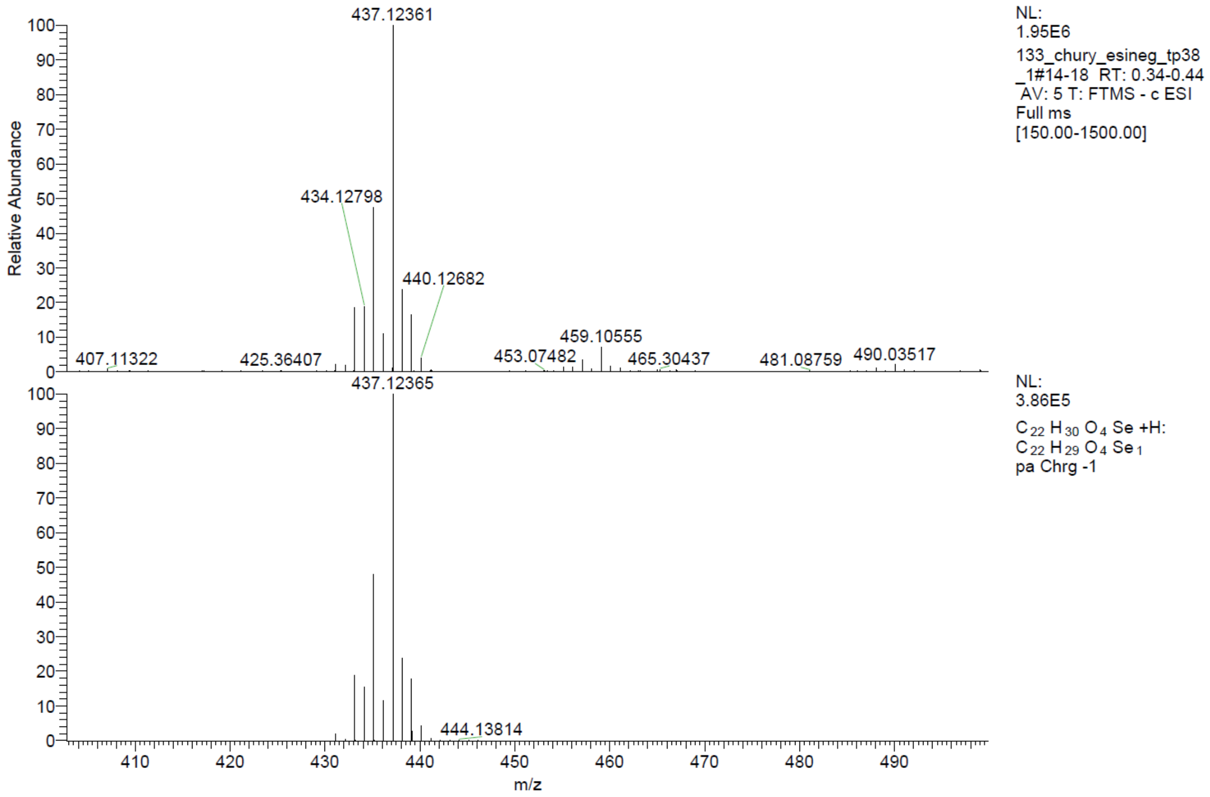

Figure S20: HRMS spectrum of compound **5** in negative mode

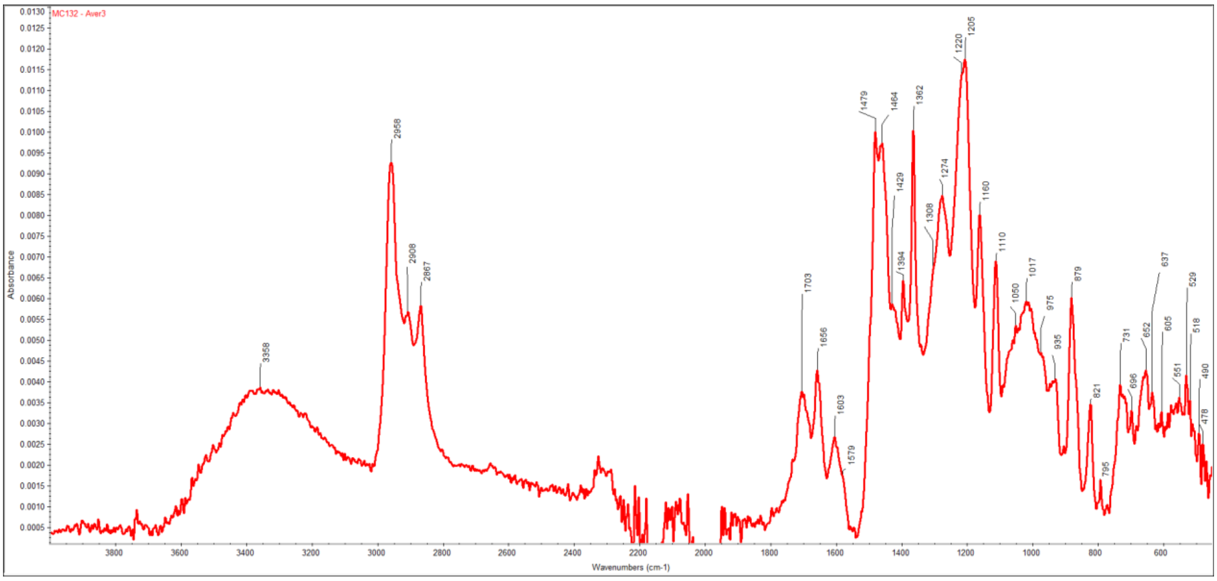

Figure S21: IR spectrum of compound **5**

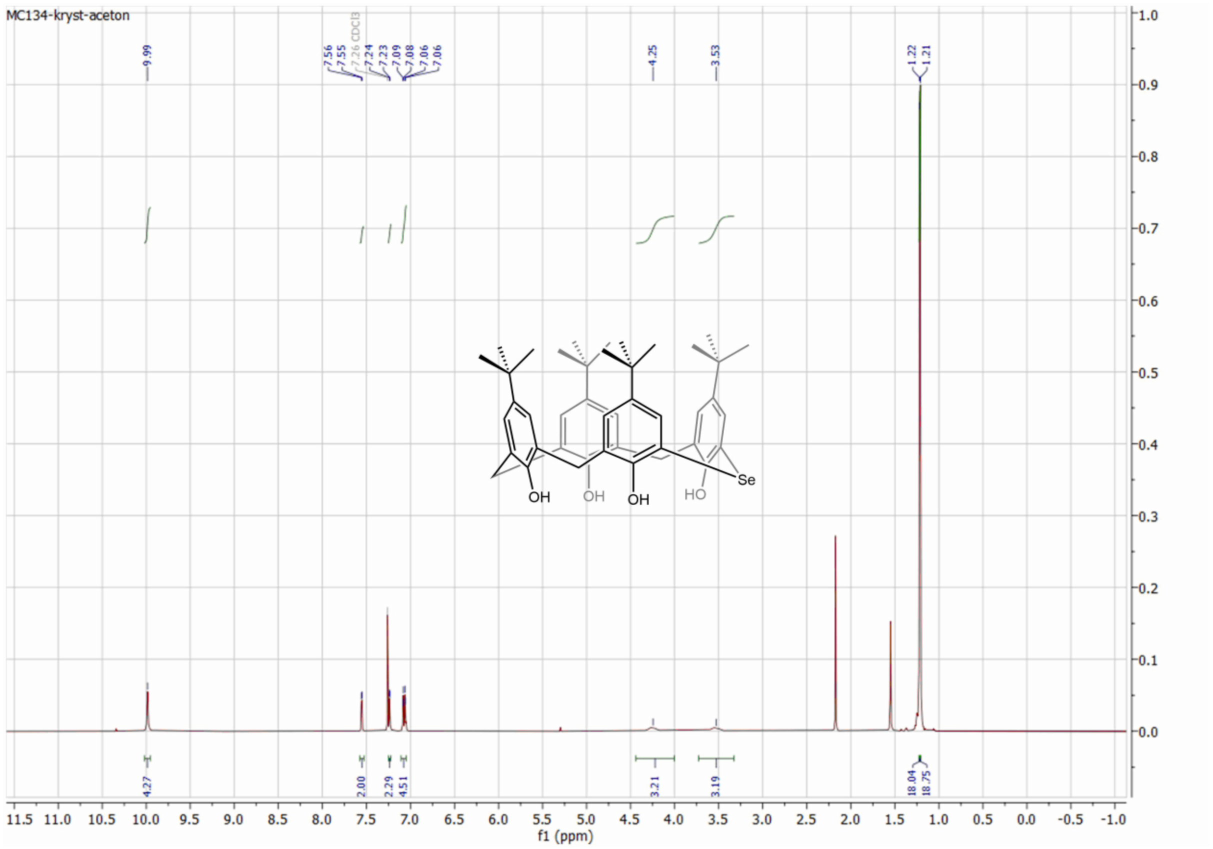

Figure S22: ^{1}H NMR spectrum of compound **8** in $CDCl_3$ at 25°C

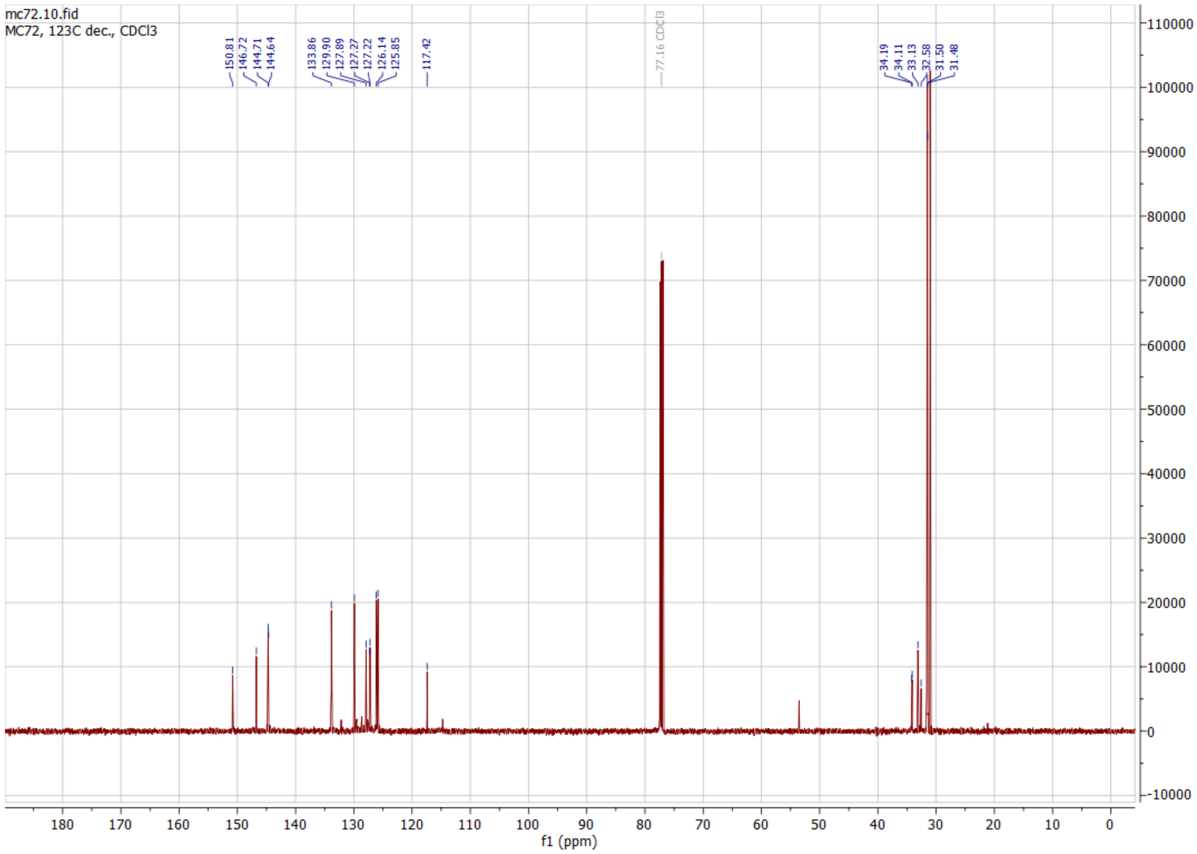

Figure S23: $^{13}C\{^{1}H\}$ NMR spectrum of compound **8** in $CDCl_3$

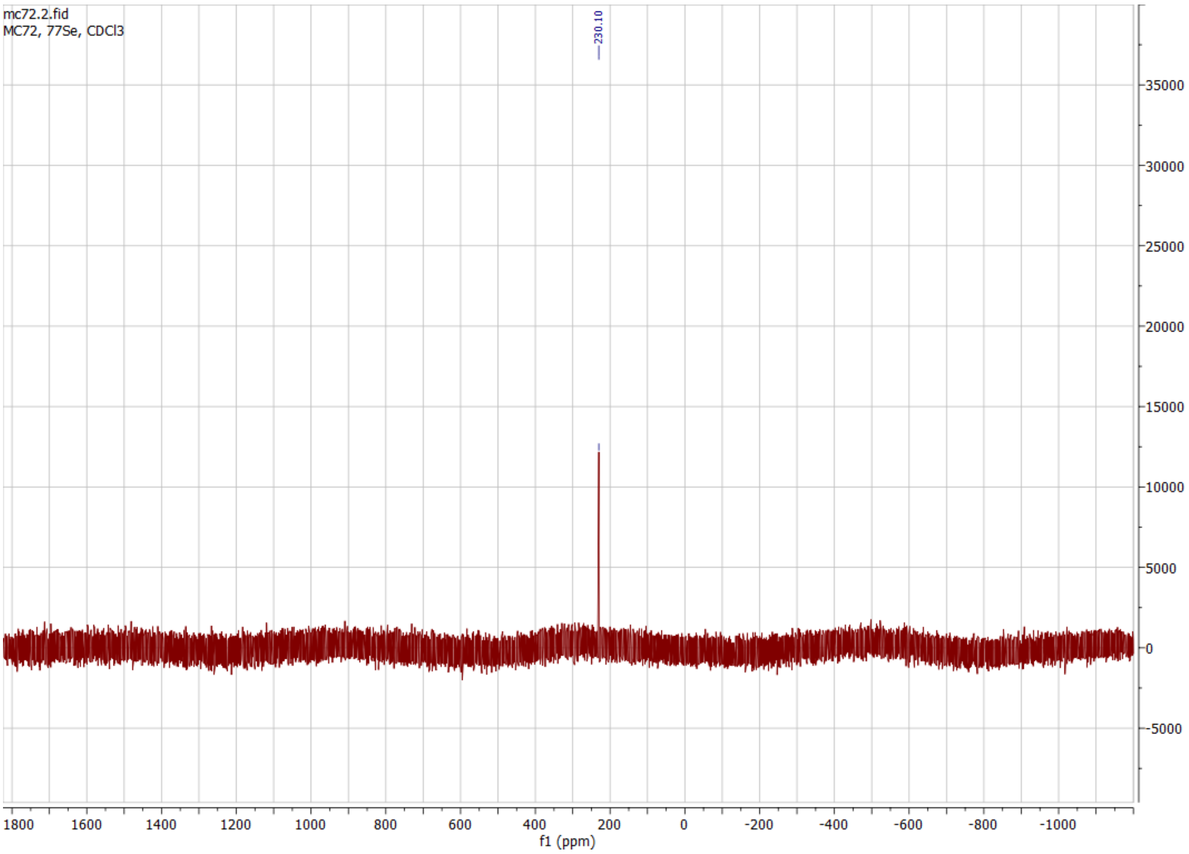

Figure S24: ^{77}Se NMR spectrum of compound **8** in $CDCl_3$

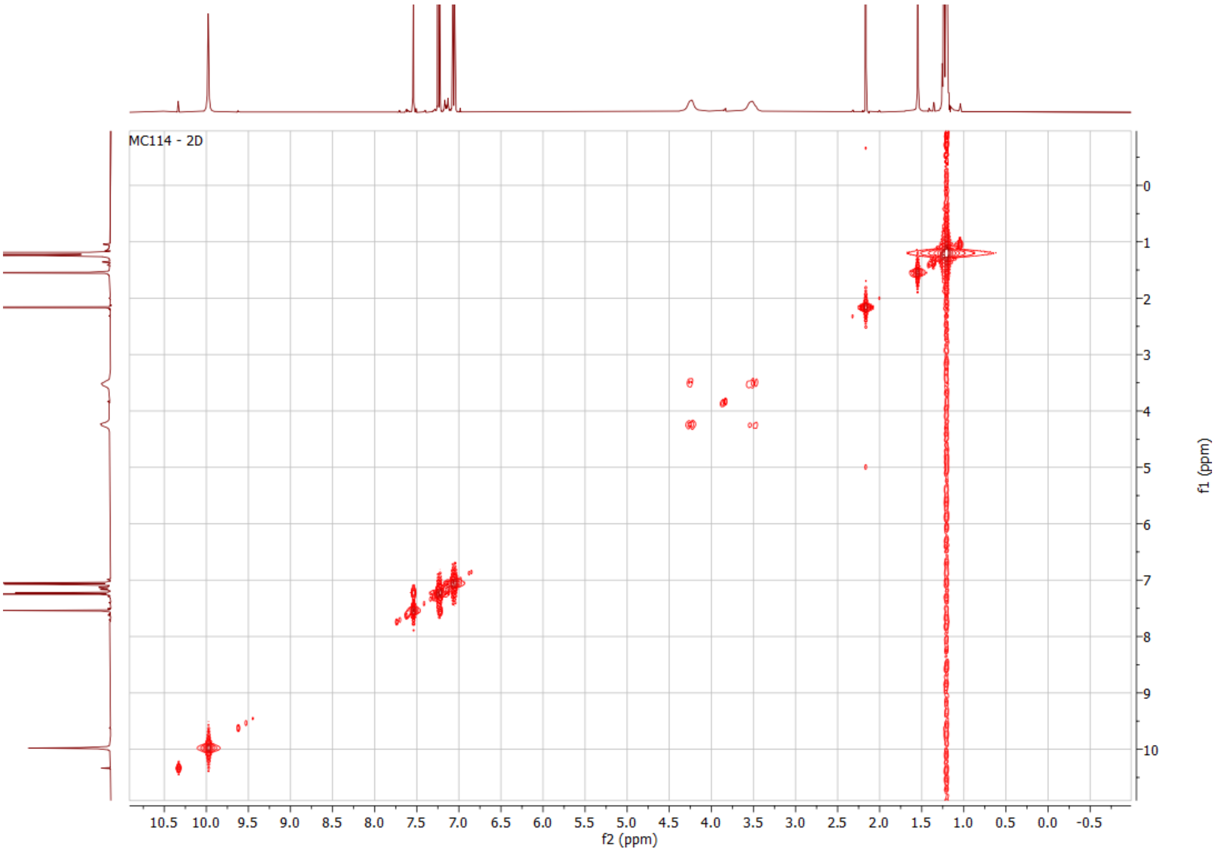

Figure S25: COSY NMR spectrum of compound **8** in $CDCl_3$

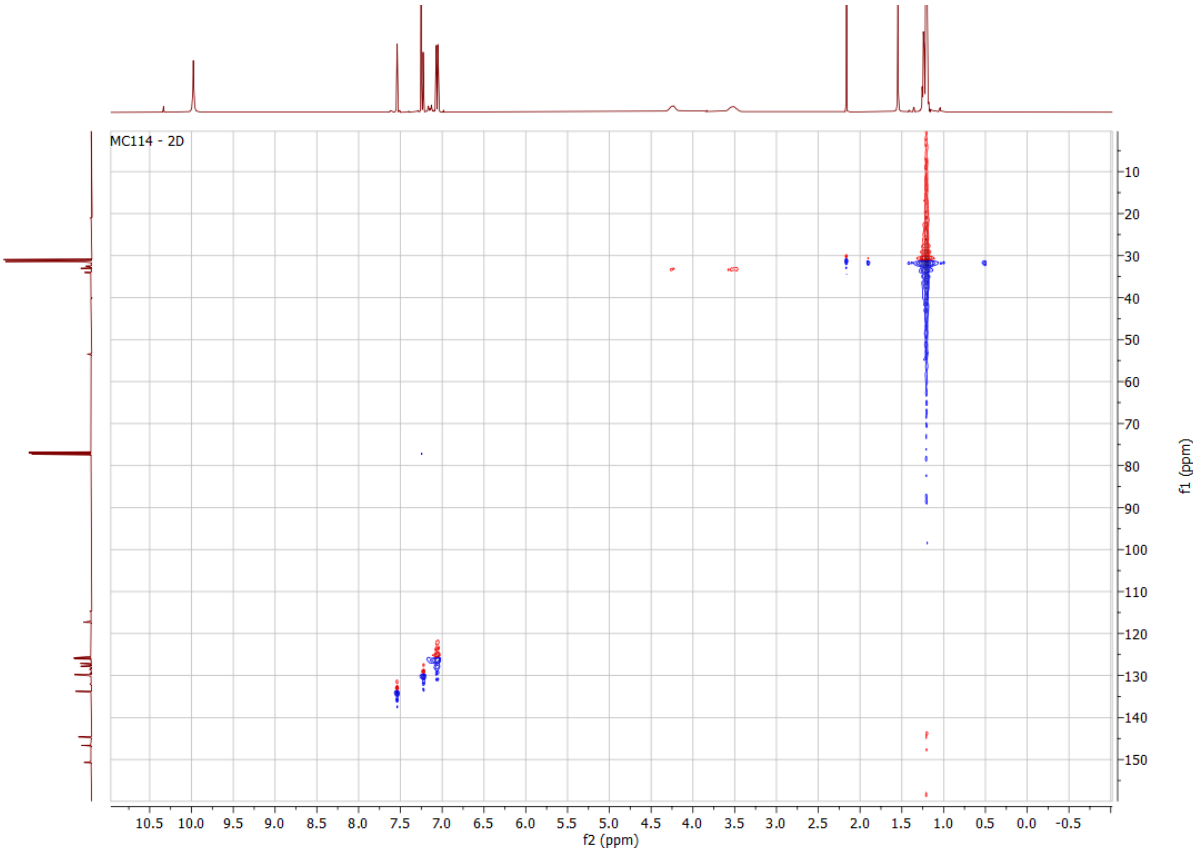

Figure S26: HSQC NMR spectrum of compound **8** in $CDCl_3$

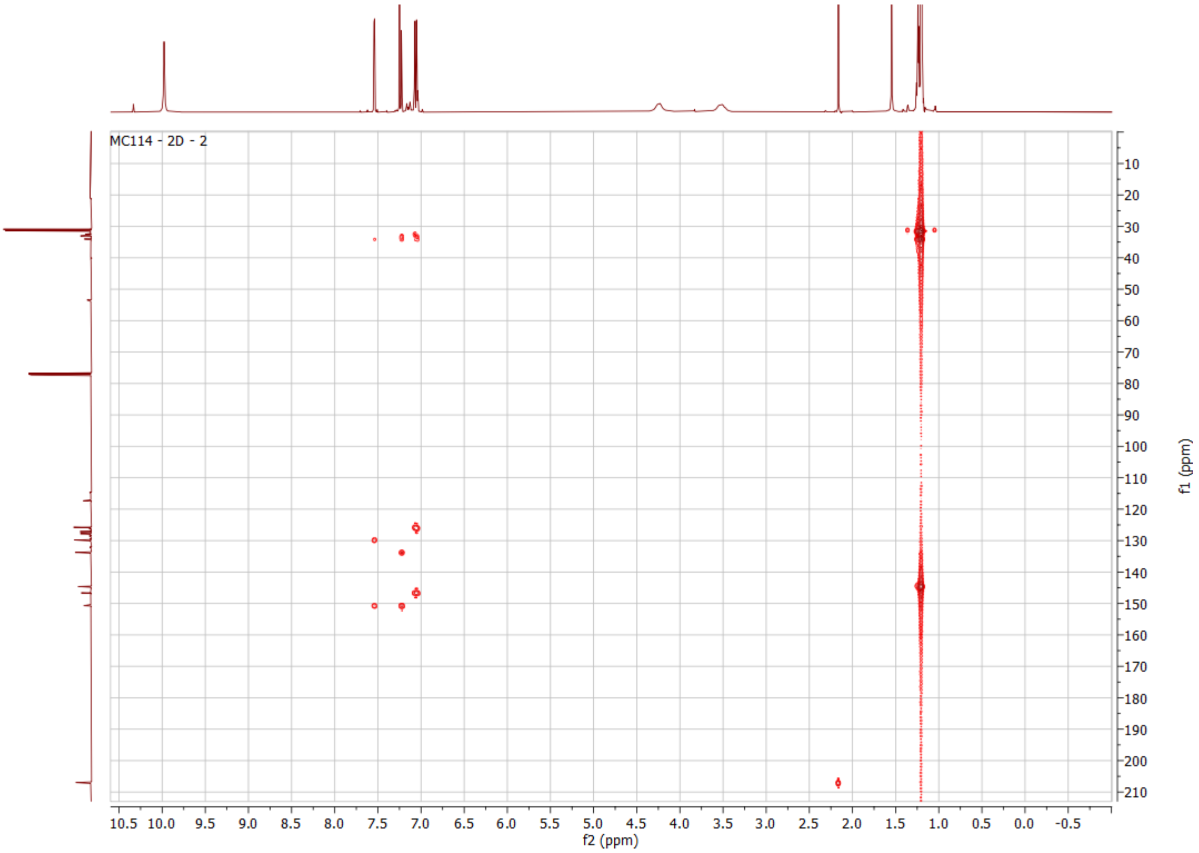

Figure S27: HMBC NMR spectrum of compound **8** in $CDCl_3$

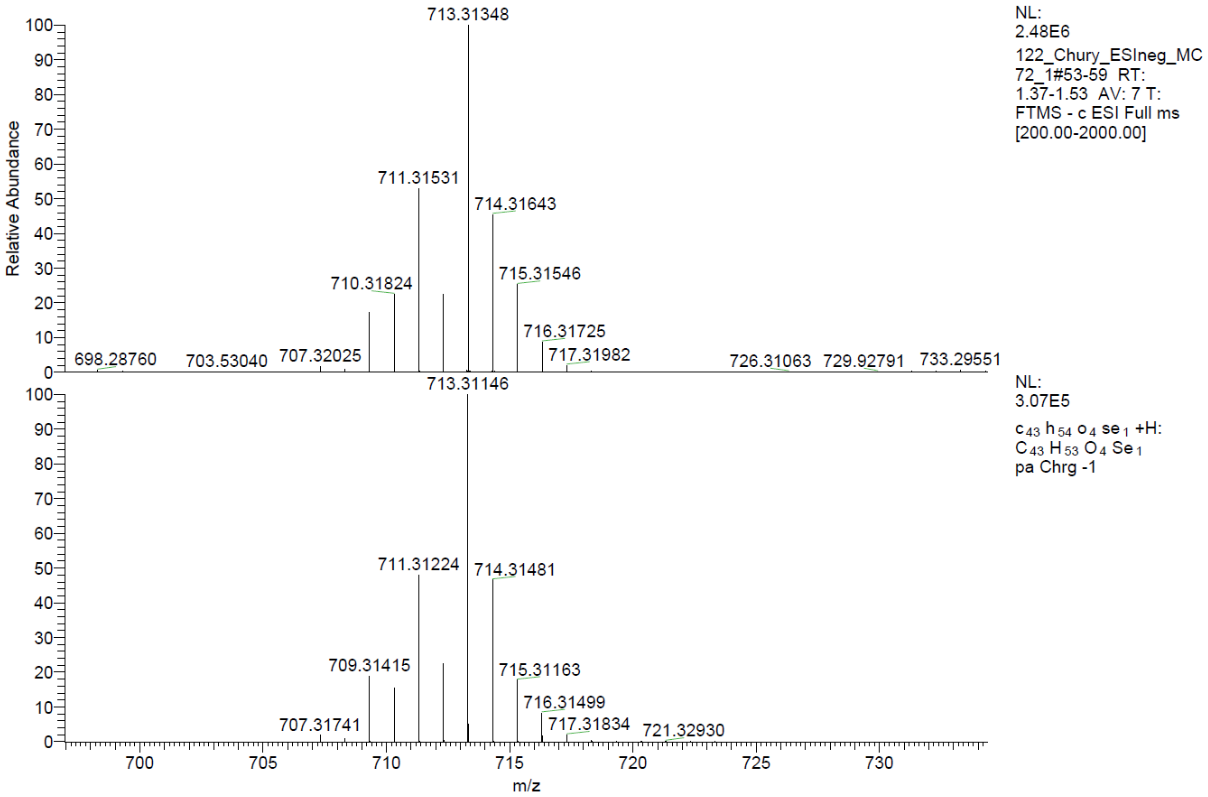

Figure S28: HRMS spectrum of compound **8**

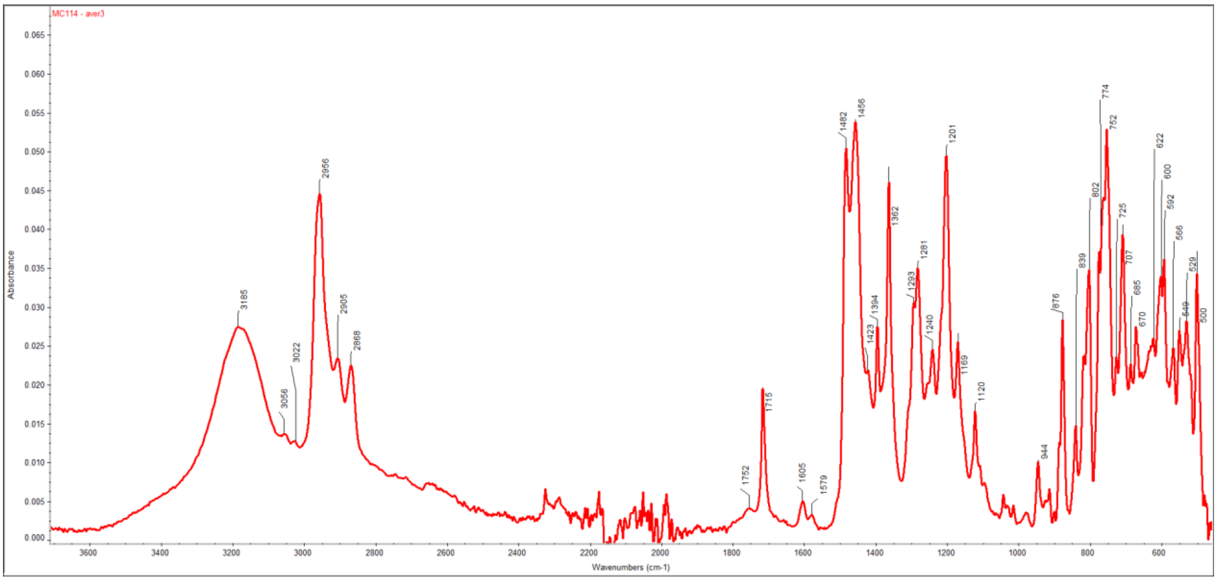

Figure S29: IR spectrum of compound **8**

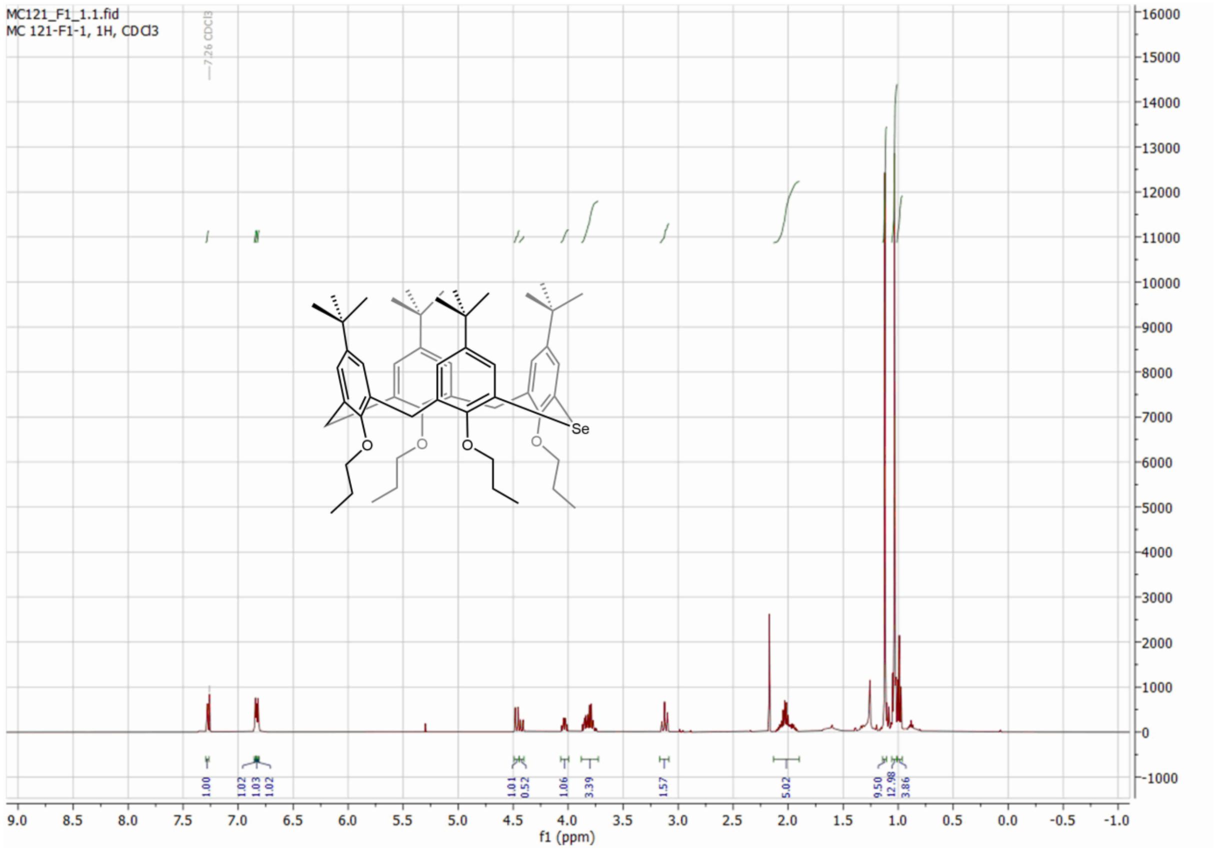

Figure S30: ^{1}H NMR spectrum of compound **9** in $CDCl_3$

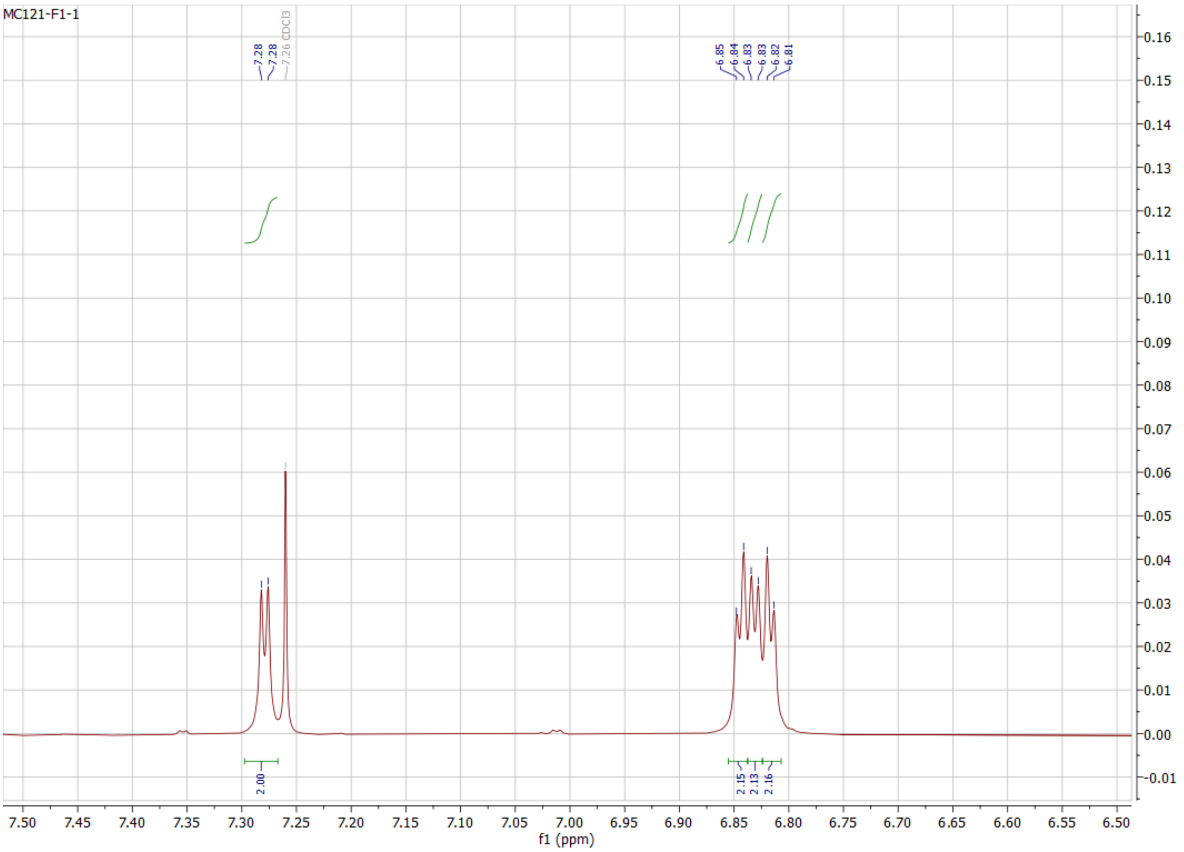

Figure S31: ^{1}H NMR spectrum of compound **9** in $CDCl_3$ – aromatic section

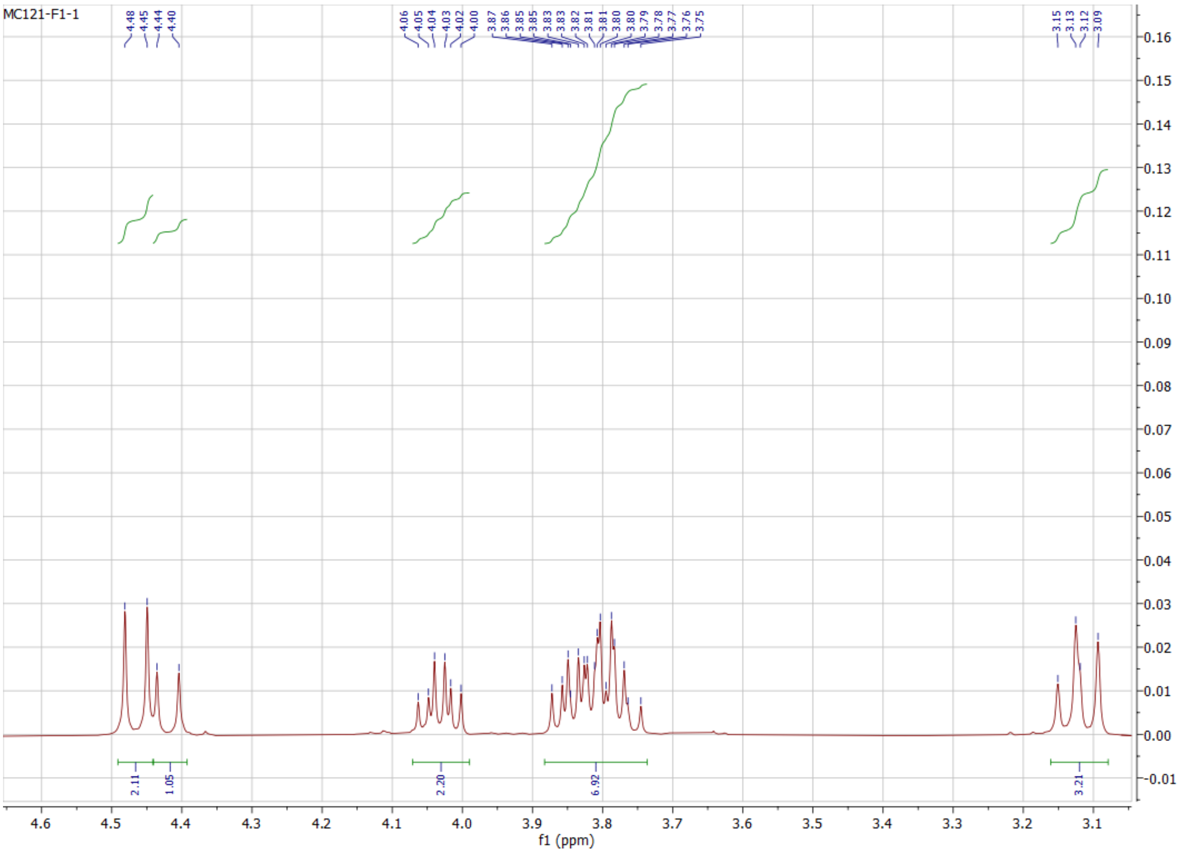

Figure S32: ^{1}H NMR spectrum of compound **9** in $CDCl_3$ – methylene bridges section

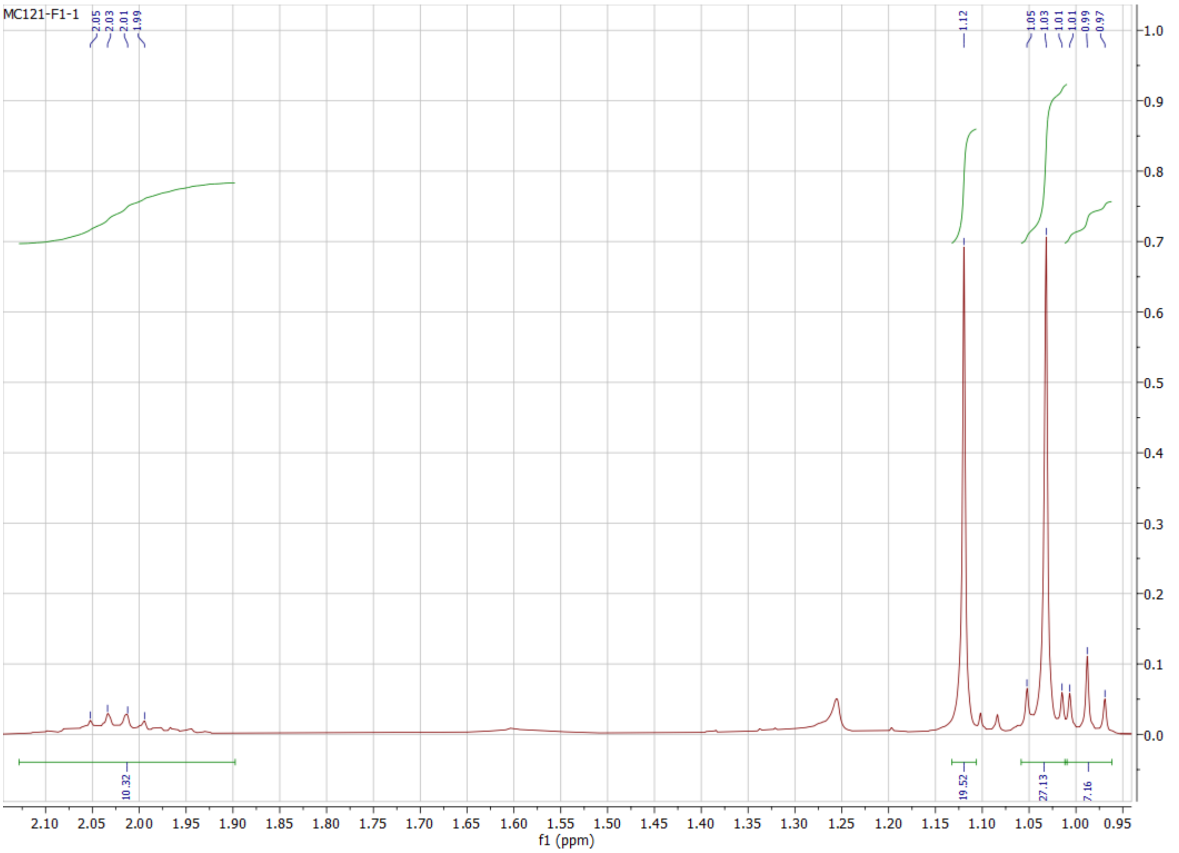

Figure S33: ^{1}H NMR spectrum of compound **9** in $CDCl_3$ – aliphatic section

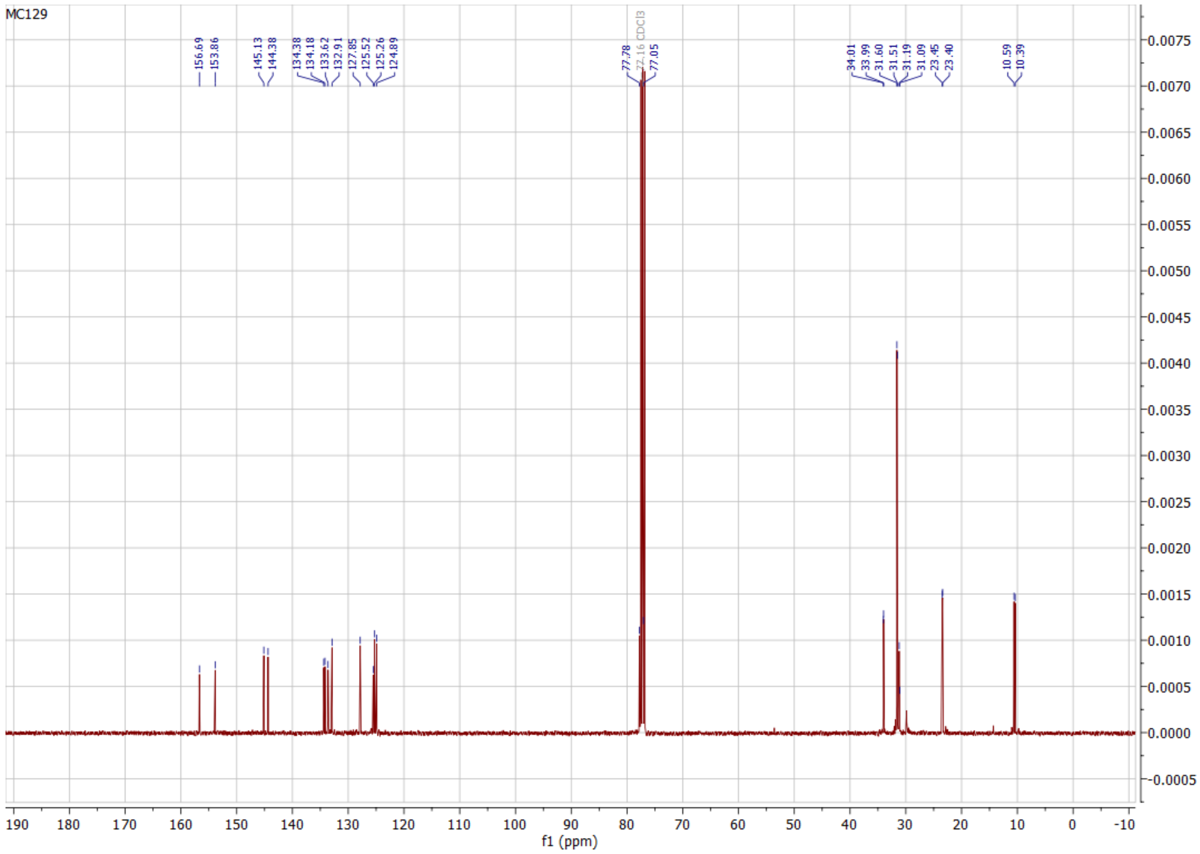

Figure S34: $^{13}C\{^{1}H\}$ NMR spectrum of compound **9** in $CDCl_3$

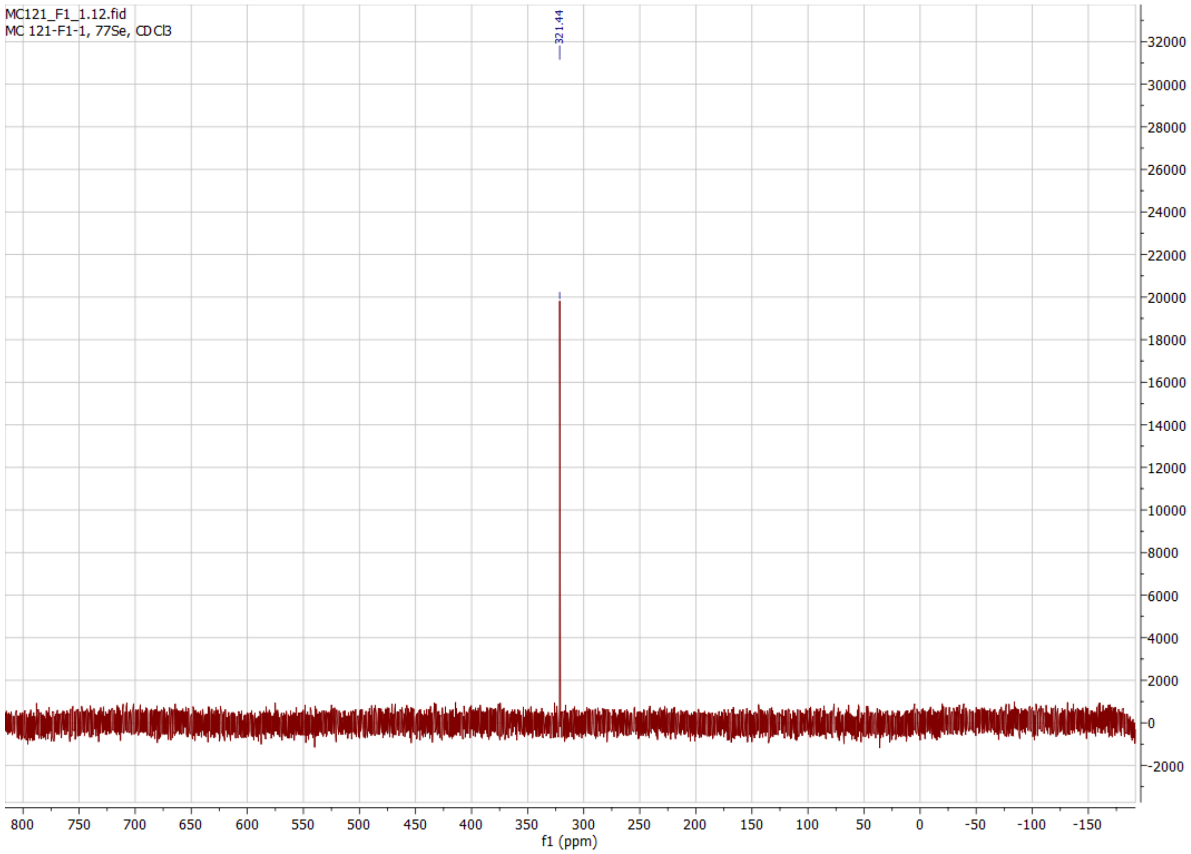

Figure S35: ^{77}Se NMR spectrum of compound **9** in $CDCl_3$

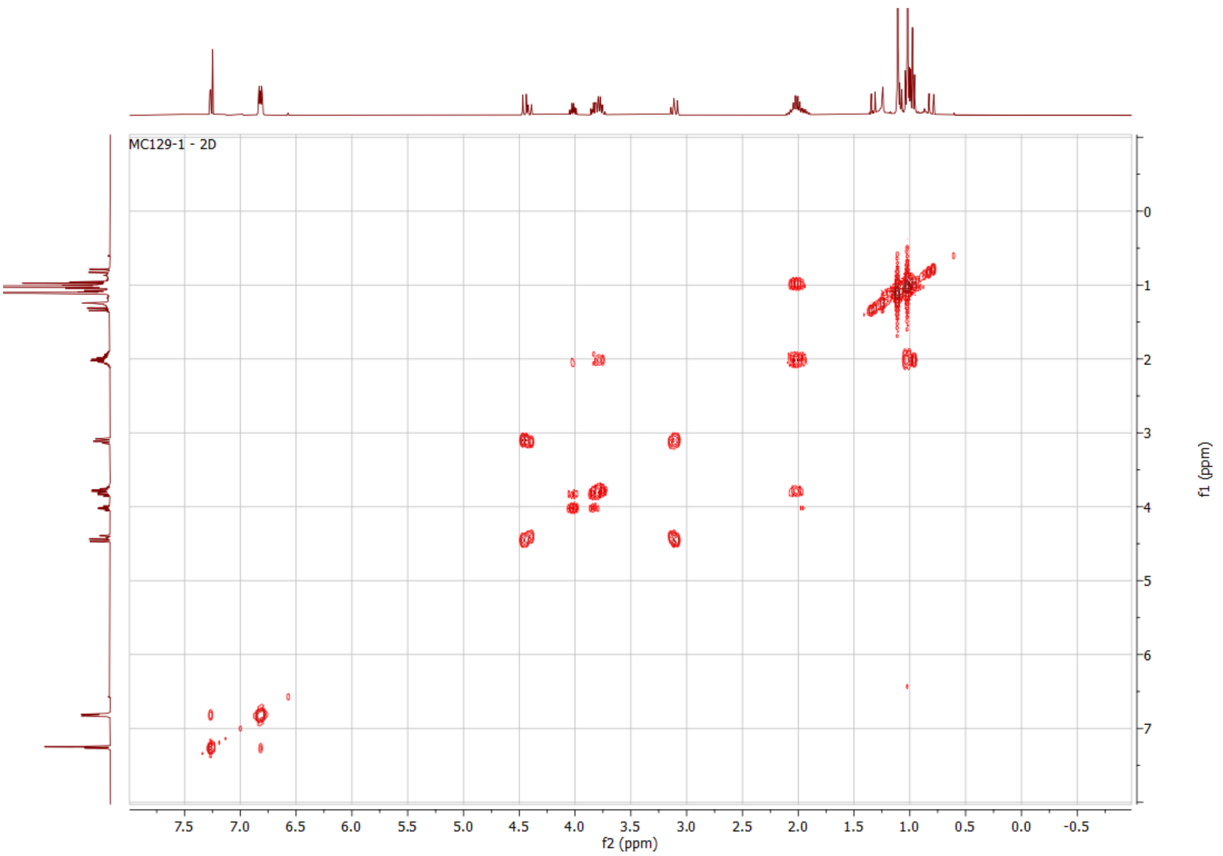

Figure S36: COSY NMR spectrum of compound **9** in $CDCl_3$

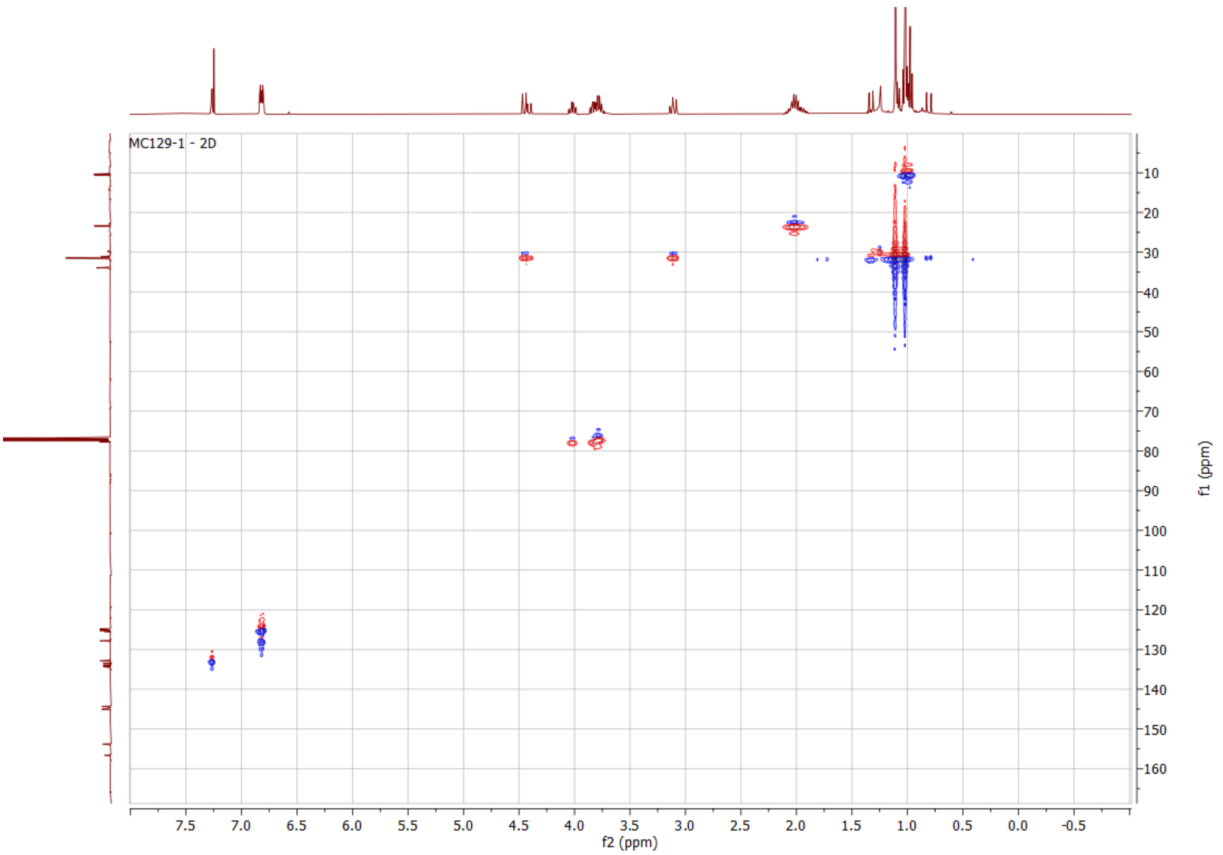

Figure S37: HSQC NMR spectrum of compound **9** in $CDCl_3$

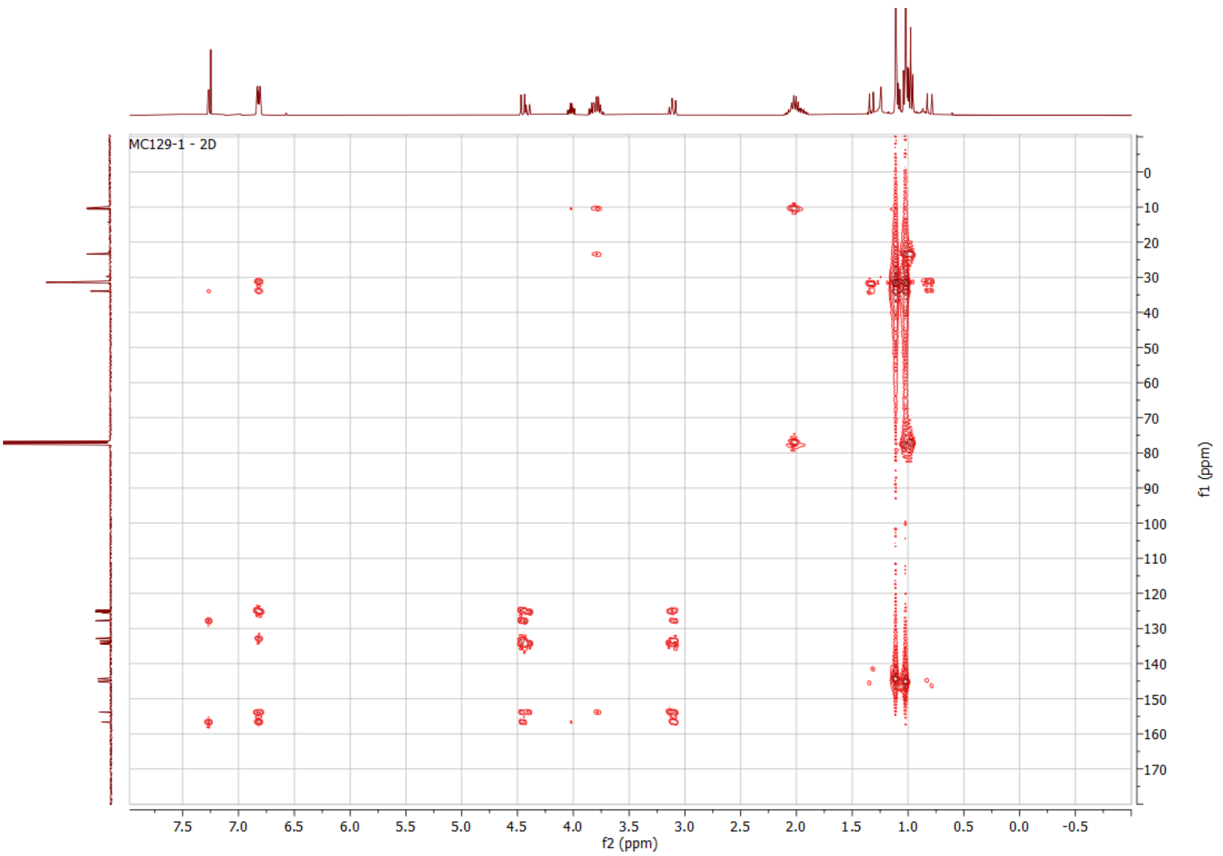

Figure S38: HMBC NMR spectrum of compound **9** in $CDCl_3$

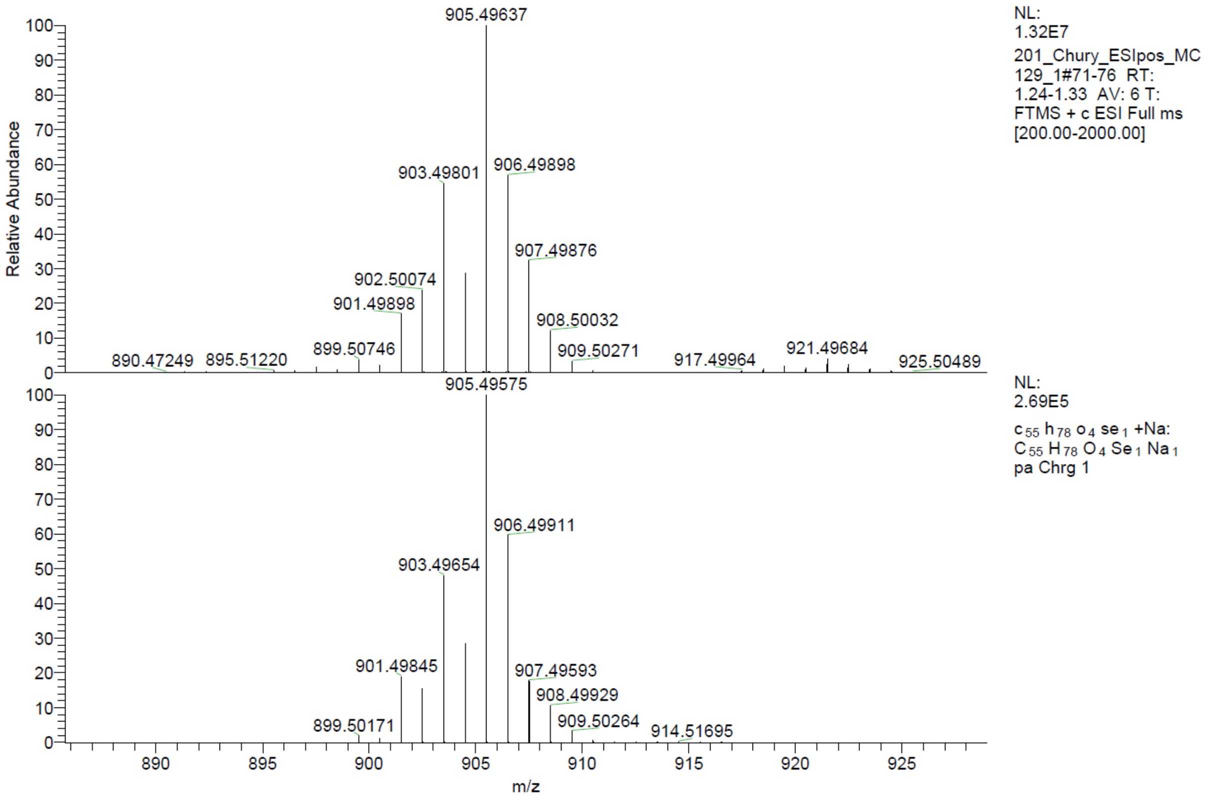

Figure S39: HRMS spectrum of compound **9**

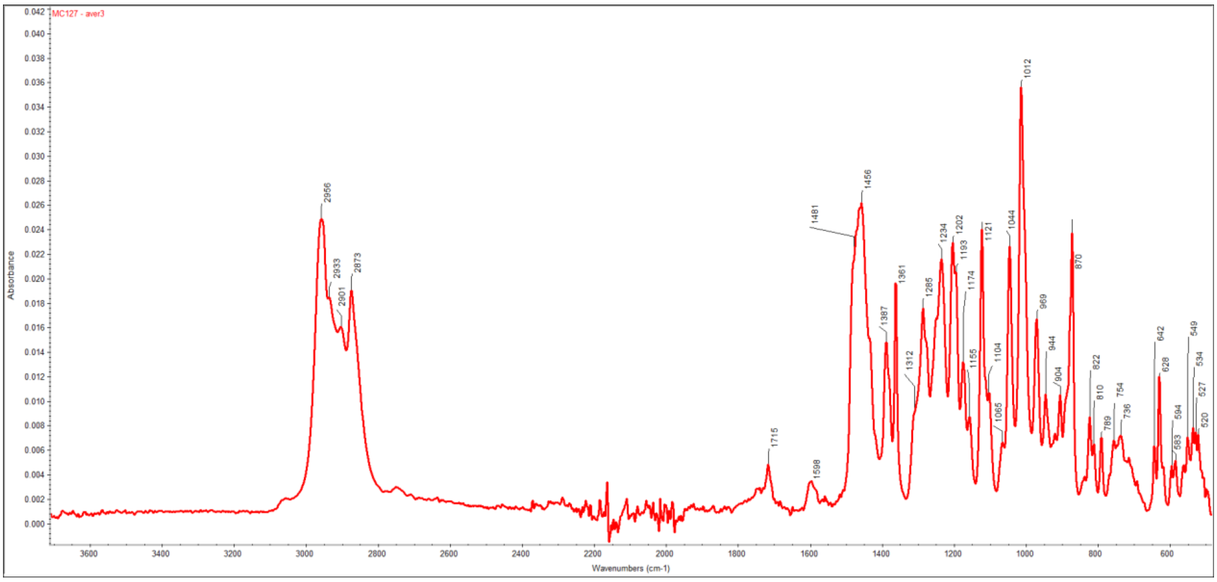

Figure S40: IR spectrum of compound **9**

4. Variable temperature NMR spectra

Variable temperature studies of **8** were performed on Bruker Avance III 500 (1H:500.13 MHz, Bremen, Germany). The dynamic behaviour of methylene bridges was followed in the temperature range 263 – 353 K in $C_2D_2Cl_4$. The obtained spectra were analysed in Dynamic NMR Models (dNMR) in the TopSpin (version 4.5.0) software in order to obtain the rate constant *k* for each temperature and calculate thermodynamic parameters. At the lowest temperature, the system provided two separate sets of signals for axial and equatorial hydrogen atoms, each of them containing contribution of three methylene bridges in the ratio 2:1 due to the symmetry of the molecule. At higher temperature, the signals of corresponding axial and equatorial protons collapsed in two lines preserving the original ratio 2:1. The chemical exchange of 2 symmetrical CH_2 groups and the residual one were fitted independently providing two rate constants k_1 and k_2. Two restrains were applied, the intensity of fitted systems were kept in the approximate ratio 2:1 due to the intensity ratio of both systems and $k_1 \cong k_2$ as both describe the same dynamic process and therefore should be equal. The dynamic behaviour was simulated in the chemical shift range 5.0 – 3.0 ppm. The agreement between the experimental and fitted spectra is depicted in the **Figure S41**. The obtained k_1 and k_2 values enabled determination of thermodynamic parameters and the activation free energy of the *cone-cone* interconversion using Eyring equations:

$$k = \frac{k_B T}{h} e^{-\frac{\Delta G^{\ddagger}}{RT}}$$

where k_B is the Boltzmann constant, *R* the gas constant and *h* the Planck constant.

The obtained values of both rate constants k_1 and k_2 and of the final overlap of the model and the corresponding experimental spectrum for each temperature are listed in **Table S5**. The dependence of ln(*k*/T) on 1/T served for determination of enthalpic parameters, **Figure S42** shows this dependence calculated for k_1 representing the major system of the two equivalent CH_2 bridges. **Table S6** summarized the obtained thermodynamic parameters for dynamic exchange of the two *cone* conformations of **8** calculated both rate constants k_1 and k_2. The final values of activation free energy $\Delta G^{\ddagger}$ were calculated for 300 K and are in good agreement.

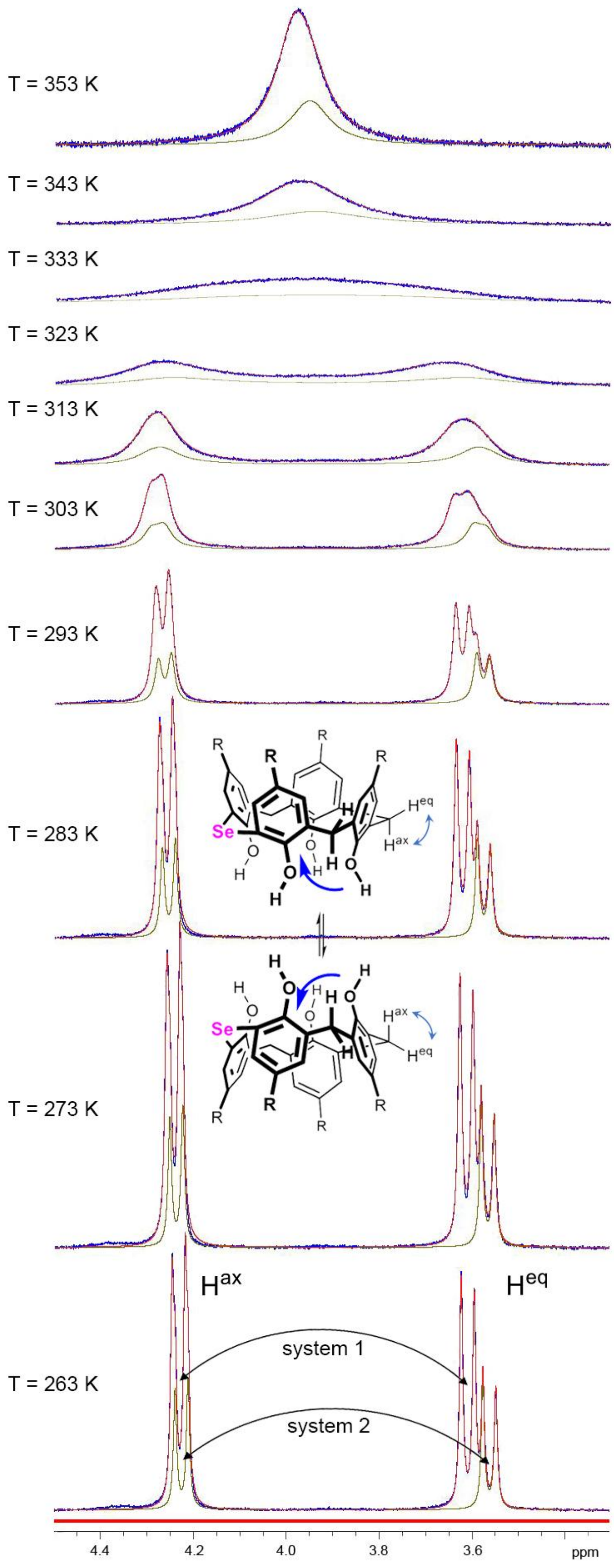

Figure S41: Spectra of the variable temperature study performed in $C_2D_2Cl_4$; experimental spectrum (blue), a sum of both simulated systems (red), a contribution of the minor system 2 (green).

Table S5: The obtained values of rate constants k_1 and k_2 for each temperature and of the final overlap of the model and the corresponding experimental spectrum.

T (K)	k_1 (Hz)	k_2 (Hz)	Overlap (%)
263	-	-	95.0
273	2.2	1.4	94.9
283	4	3.7	95.4
293	13.2	14.8	96.2
298	29.3	25.1	96.2
303	50.6	51.7	96.5
313	125.6	123	96.3
323	346	344	96.1
333	853	836	95.9
343	1974	1845	95.3
353	4058	4657	94.8

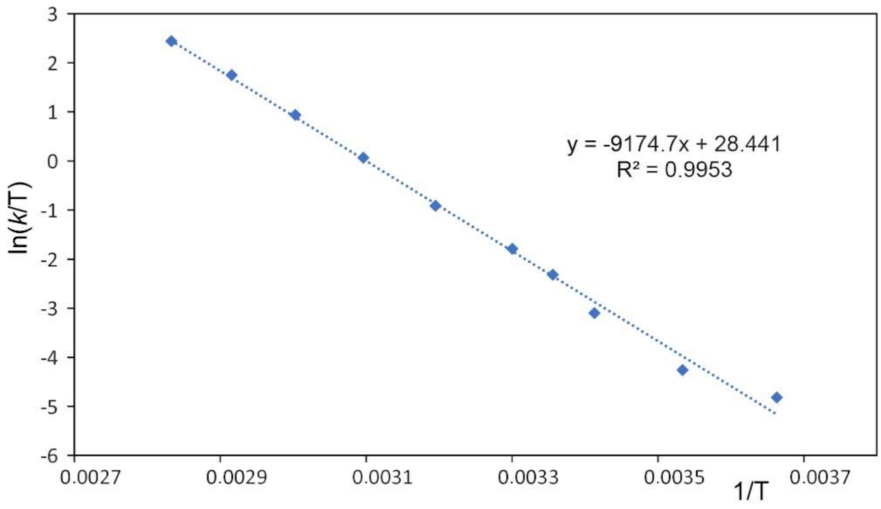

Figure S42: The dependence of ln(k/T) on 1/T calculated for k_1 (the major system, two equivalent CH_2 bridges).

Table S6: The obtained thermodynamic parameters for both exchanging systems

system	$\Delta H^‡$ (kJ/mol)	$\Delta S^‡$ (J/K·mol)	$\Delta G^‡_{300K}$ (kJ/mol)	$\Delta G^‡_{300K}$ (kcal/mol)
2 x CH_2 (k_1)	76.3	38.9	64.6	15.4
1 x CH_2 (k_2)	79.3	48.4	64.8	15.5

The second variable temperature study of **8** was focused on a dynamic behaviour of phenolic OH groups and was performed in CD_2Cl_2 in the temperature range 173 – 253 K. The obtained spectra were also analysed in Dynamic NMR Models (dNMR) in the TopSpin (version 4.5.0) software which provided the rate constant *k* for each temperature. At the lowest temperature, the system provided three resolved OH signals in the ratio 2:1:1 as two OH resonances accidentally occurred at a similar chemical shift 9.70 ppm. The two remaining signals were located at 9.32 and 9.06 ppm. Increasing the temperature, a chemical exchange between two systems was observed. One system consists of a signal at 9.70 and 9.32 ppm and the second one contains the other signal at 9.70 and the residual signal at 9.06 ppm. At elevated temperature, the pairs of signals collapse into two signals of the equal intensity. Again, the chemical exchange of 2 two systems were fitted independently providing two rate constants k_1 and k_2, both describing the same dynamic process. The dynamic behaviour was simulated in the chemical shift range 10.5 – 8.5 ppm keeping $k_1 \cong k_2$. The agreement between the experimental and fitted spectra is depicted in the **Figure S43** together with a schematic depiction of the *flip-flow* dynamics of OH groups changing the orientation of circular hydrogen bond which is manifested in NMR spectra by a chemical exchange between two pairs of signals. Unfortunately, all attempts to assigned individual OH signals failed. The activation free energy of the *flip-flop* motion of OH groups in molecule **8** was then calculated using Eyring equations. The obtained values of the rate constants k_1 and k_2 and of the final overlap of the model and the experiment for each temperature are listed in **Table S7**. **Figure S44** shows the dependence of ln(*k*/T) on 1/T calculated for k_1 and **Table S8** summarized the obtained thermodynamic parameters provided by both simulated systems. The final values of activation free energy $\Delta G^{\ddagger}$ were calculated for 300 K and are in good agreement for both systems.

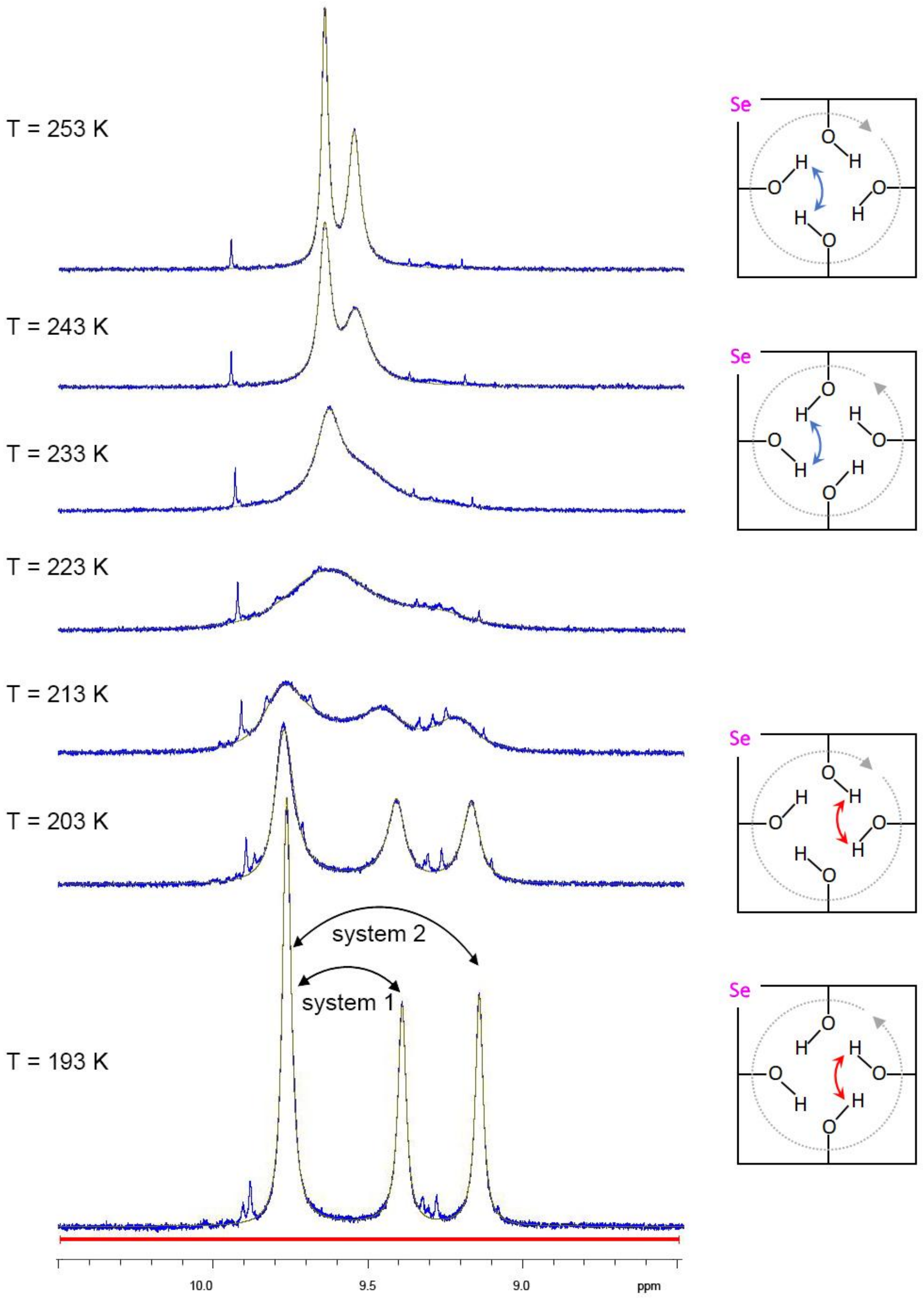

Figure S43: Spectra of the variable temperature study performed in CD_2Cl_2 (left); experimental spectrum (blue), a sum of both simulated systems (green). A schematic depiction of the *flip-flow* dynamics of OH groups when changing the orientation of circular hydrogen bond (right). The pairs of two exchanging protons are indicated by an arrow.

Table S7: The obtained values of rate constants k_1 and k_2 for each temperature and of the final overlap of the model and the corresponding experimental spectrum.

T (K)	k_1 (Hz)	k_2 (Hz)	Overlap (%)
193	24.5	22	95.3
203	80.6	79.5	94.9
213	244.8	222.3	95.3
223	518.3	501.1	95.1
233	1219	1198	95.3
243	2575	2881	94.9
253	5500	5500	94.9

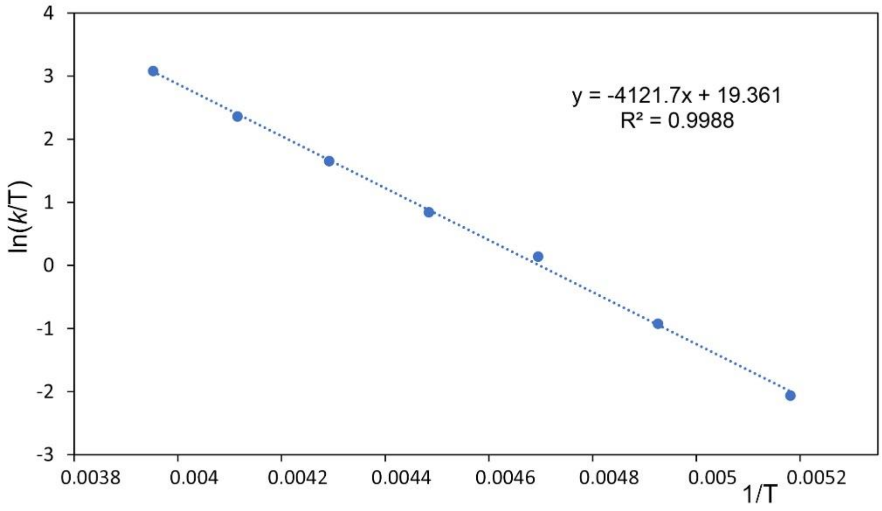

Figure S44: The dependence of ln(k/T) on 1/T calculated for k_1.

Table S8: The obtained thermodynamic parameters for both exchanging systems of phenolic OH groups.

system	$\Delta H^{\ddagger}$ (kJ/mol)	$\Delta S^{\ddagger}$ (J/K·mol)	$\Delta G^{\ddagger}_{300K}$ (kJ/mol)	$\Delta G^{\ddagger}_{300K}$ (kcal/mol)
9.70 → 9.32 (k_1)	34.3	-36.6	45.2	10.8
9.70 → 9.06 (k_2)	35.2	-32.5	45.0	10.7

5. Crystallographic data

Single crystals were obtained by slow evaporation of solutions of compound **8** or **9** in chloroform.

Compound 8

M = 713.86 g.mol^{-1}, tetragonal system, space group *P*4/*n*, *a* = 13.0522 (4) Å, *c* = 12.8766 (6) Å, *Z* = 2, *V* = 2193.66 (16) Å^3, D_c = 1.081 g.cm^{-3}, μ(Mo*Kα*) = 0.89 mm^1, crystal dimensions of 0.55 × 0.47 × 0.21 mm. Data were collected at 180 (2) K on a Bruker D8 Venture Photon CMOS diffractometer with Incoatec microfocus sealed tube Mo-*Kα* radiation. The structure was solved by charge flipping methods[S4] and anisotropically refined by full matrix least squares on *F* squared using the CRYSTALS[S5] to final value *R* = 0.082 and *wR* = 0.246 using 2746 independent reflections (ϑ_{max} = 28.46°), 122 parameters and 2 restrains. The hydrogen atoms bonded to carbon atoms were placed in calculated positions refined with a riding constrains. The hydrogen atom bonded to oxygen was refined with restrained geometry. The disordered selenium and methylene bridge positions were found in difference electron density maps and refined without restrictions. To keep the molecular composition the occupancy ratio was fixed to 0.25:0.75 (Se:CH_2). MCE[S6] was used for visualization of electron density maps. Highly disordered solvent was observed in the selenacalix[4]arene cavity. The solvent disorder was further complicated by the presence of fourfold axis; therefore, we have decided to remove the solvent from the structure model and calculate its contribution to structure factors using PLATON squeeze[S7]. The structure was deposited into Cambridge Structural Database under number CCDC 2489379.

Compound 9

M = 882.18 g.mol^{-1}, trigonal system, space group $P3_121$, *a* = 12.98342 (12) Å, *c* = 25.4914 (3) Å, *Z* = 3, *V* = 3721.37 (7) Å^3, D_c = 1.181 g.cm^{-3}, μ(Cu*Kα*) = 1.35 mm^1, crystal dimensions of 0.07 × 0.04 × 0.04 mm. Data were collected at 100 (2) K on a XtaLAB Synergy-R using Cu-*Kα* (λ = 1.54180 Å) radiation from PhotonJet-R microfocus rotating anode X-ray source and HyPix-Arc 150° HPC detector. The structure was solved by charge flipping methods[S4] and anisotropically refined by full matrix least squares on *F* squared using the CRYSTALS[S5] to final value *R* = 0.057 and *wR* = 0.168 using 5135 independent reflections (ϑ_{max} = 75.84°), 308 parameters and 58 restrains. The hydrogen atoms bonded to carbon atoms were placed in calculated positions refined with a riding constrains. disordered selenium and methylene bridge positions were found in difference electron density maps and refined with restrained ADPs. To keep the molecular composition the occupancy ratio was fixed to 0.5:0.5 (Se:CH_2). The disordered propoxy group positions were found in difference electron density maps and refined with restrained geometry and ADPs. To avoid the potential collisions of disordered positions the occupancy factors had to be fixed to 0.5:0.5. When one position is present its symmetry equivalent is absent. The transition to lower symmetry was tested; however, the disorder is clearly present even in lower $P3_1$ symmetry. MCE[S6] was used for visualization of electron density maps. The structure was deposited into Cambridge Structural Database under number CCDC 2489380.

(S4) Palatinus, L., Chapuis, G., *J. Appl. Cryst.* **2007**, *40* (4), 786-790.
(S5) Betteridge, P.W., Carruthers, J.R., Cooper, R.I., Prout, K., Watkin, D.J., *J. Appl. Cryst.* **2003**, *36*, 1487.
(S6) Rohlicek J., Husak M., *J. Appl. Cryst*. **2007**, *40* (3), 600-601.
(S7) A.L.Spek, *Acta Cryst*. **2009**, *D65*, 148-155.

6. Single crystal X-ray structures

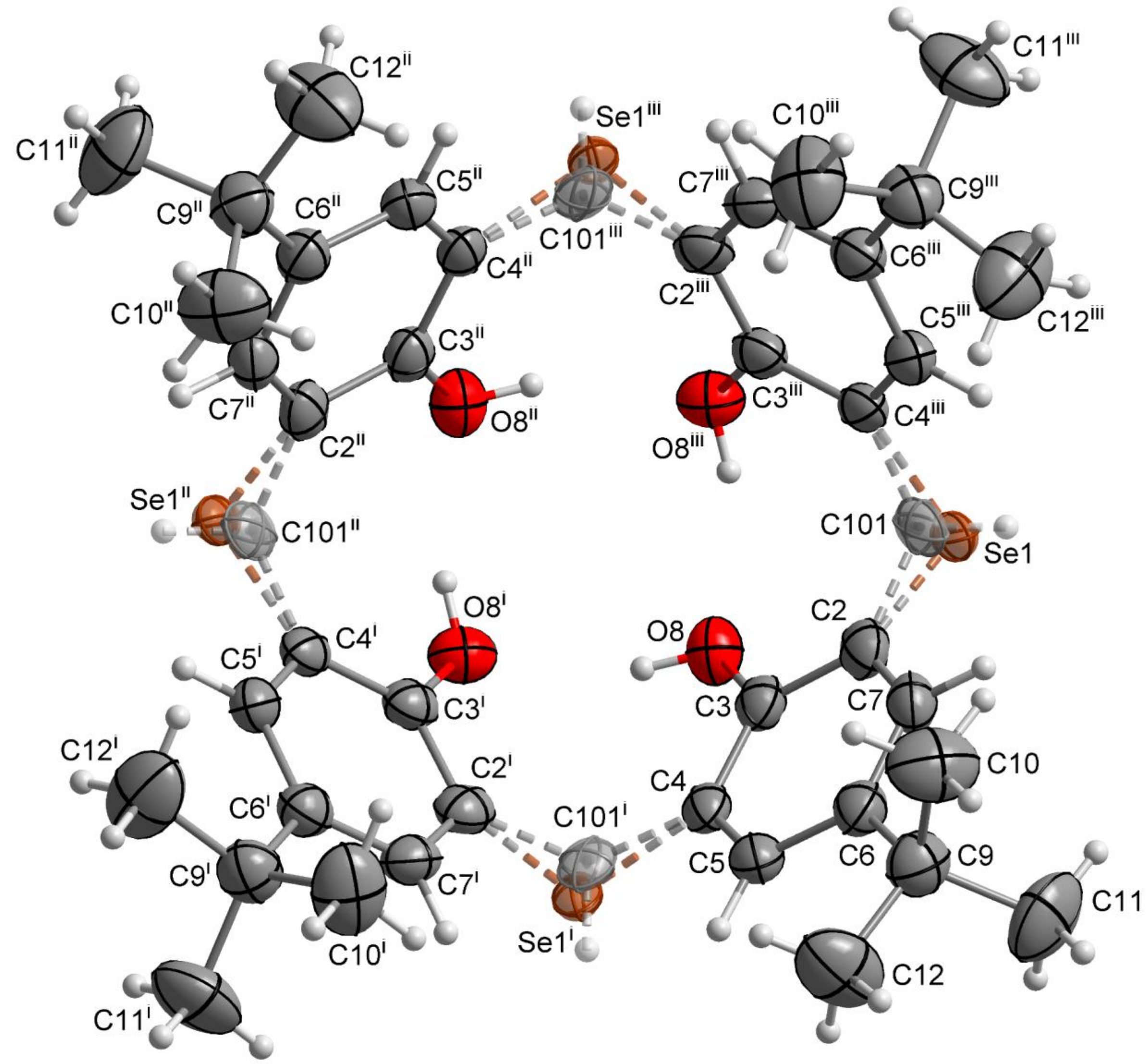

Figure S45: The numbering scheme of crystal structure **8**, with ADPs drawn at 50% probability level. The weakly occupied atoms are depicted as transparent, with dashed bonds. Symmetry codes: (*i*) *y*, 1.5-*x*, *z*; (*ii*) 1.5-*x*, 1.5-*y*, *z*; (*iii*) 1.5-*y*, *x*, *z*.

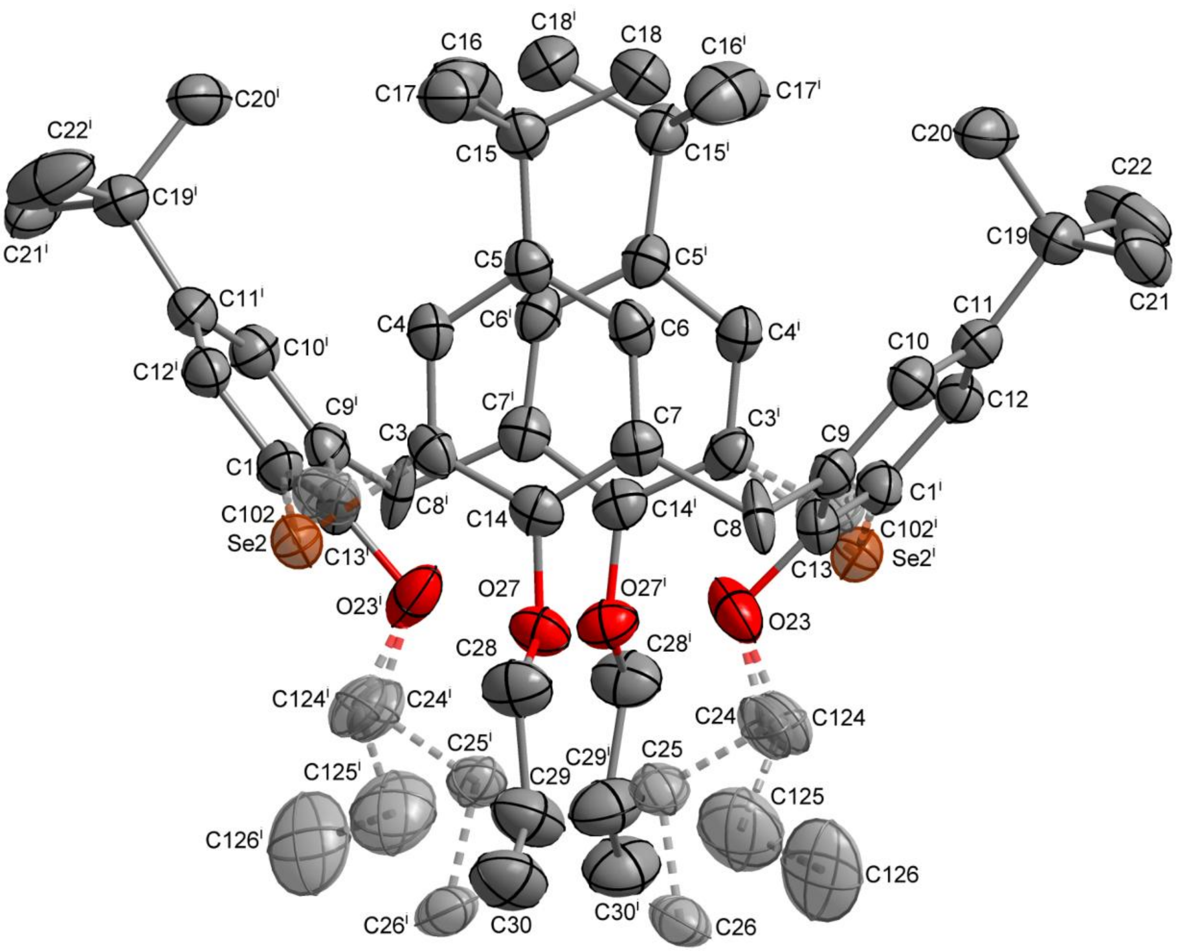

Figure S46: The numbering scheme of crystal structure **9**, with ADPs drawn at 50% probability level. The weakly occupied atoms are depicted as transparent, with dashed bonds. Hydrogen atoms were omitted for clarity. Symmetry codes: (*i*) *y*, *x*, 1-*z*.

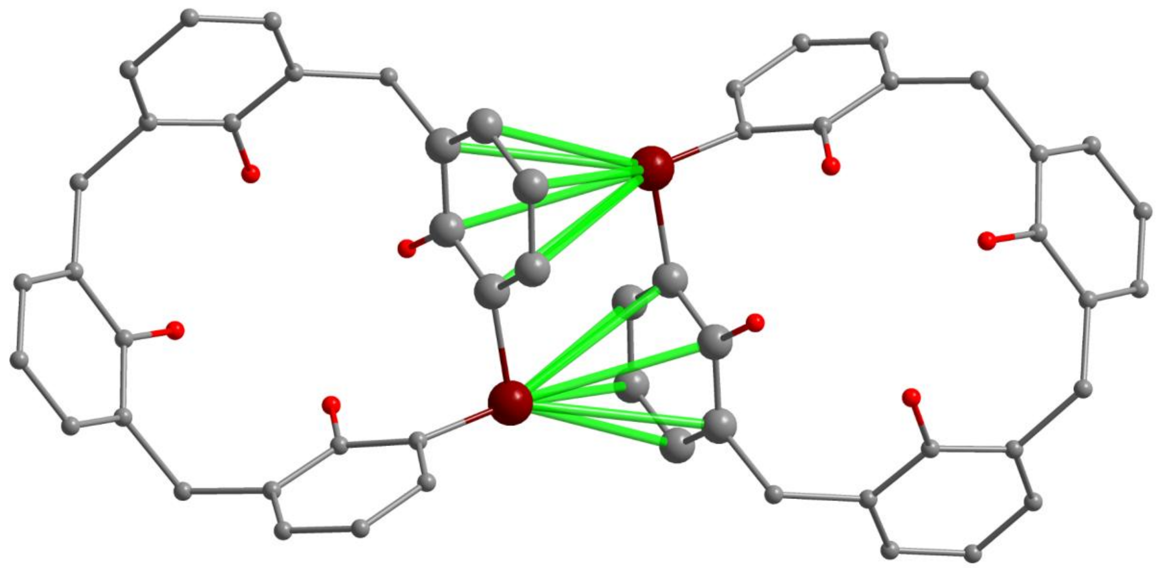

Figure S47: The structure of possible dimer formed by compound **8**, the interacting atoms were depicted as spheres of arbitrary radii. The hydrogens and *tert*-butyl groups were omitted for clarity.

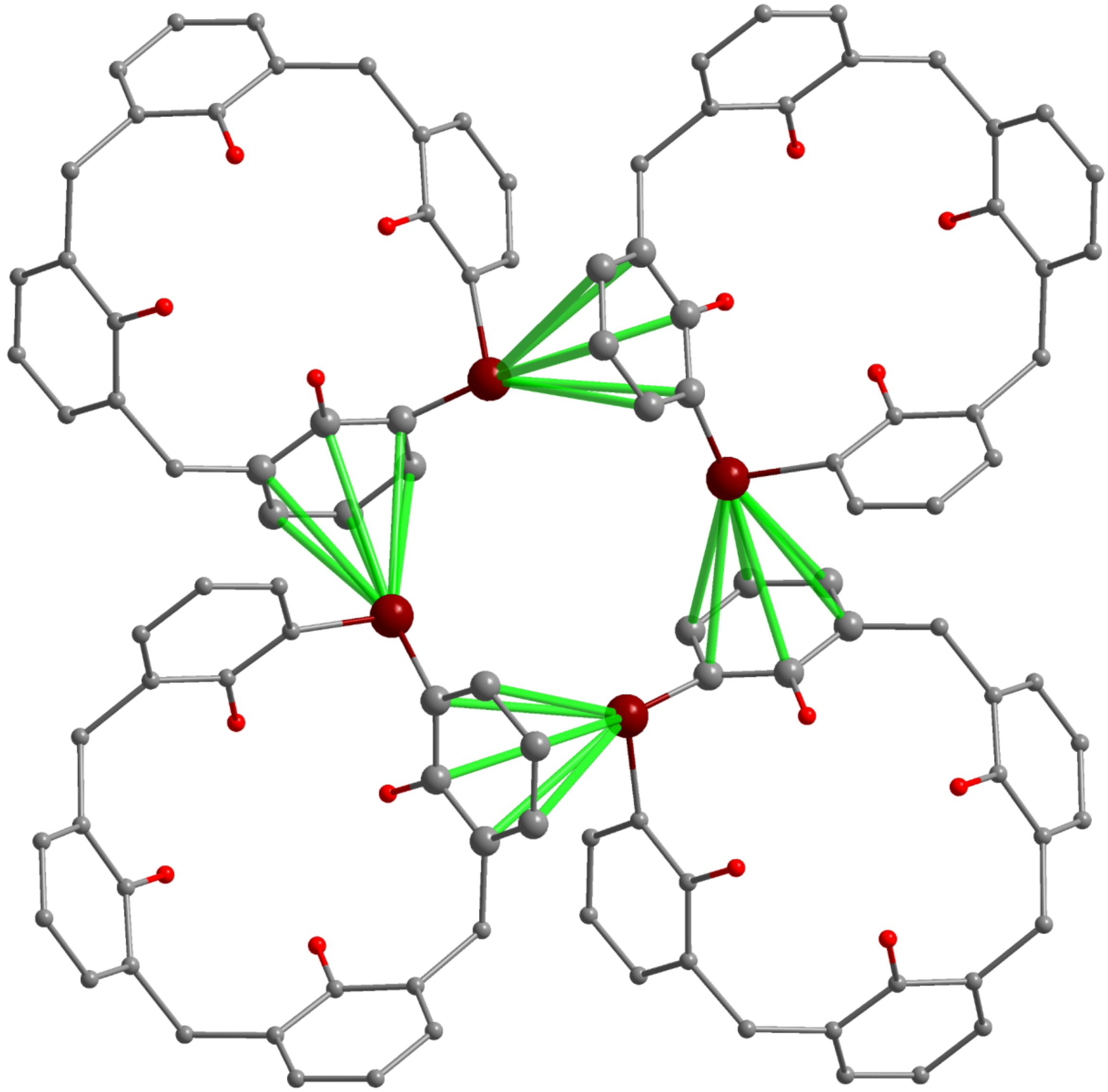

Figure S48: The structure of possible tetramer formed by compound **8**, the interacting atoms were depicted as spheres of arbitrary radii. The hydrogens and *tert*-butyl groups were omitted for clarity.
